# Modeling protected species distributions and habitats to inform siting and management of pioneering ocean industries: A case study for Gulf of Mexico aquaculture

**DOI:** 10.1371/journal.pone.0267333

**Published:** 2022-09-30

**Authors:** Nicholas A. Farmer, Jessica R. Powell, James A. Morris, Melissa S. Soldevilla, Lisa C. Wickliffe, Jonathan A. Jossart, Jonathan K. MacKay, Alyssa L. Randall, Gretchen E. Bath, Penny Ruvelas, Laura Gray, Jennifer Lee, Wendy Piniak, Lance Garrison, Robert Hardy, Kristen M. Hart, Chris Sasso, Lesley Stokes, Kenneth L. Riley

**Affiliations:** 1 NOAA/National Marine Fisheries Service, Southeast Regional Office, St. Petersburg, Florida, United States of America; 2 NOAA/National Ocean Service, National Centers for Coastal Ocean Science, Beaufort, North Carolina, United States of America; 3 NOAA/National Marine Fisheries Service, Southeast Fisheries Science Center, Miami, Florida, United States of America; 4 CSS, Inc. under contract to the National Centers for Coastal Ocean Science, National Ocean Service, NOAA, Beaufort, North Carolina, United States of America; 5 NOAA/National Marine Fisheries Service, West Coast Regional Office, Long Beach, California, United States of America; 6 NOAA/National Marine Fisheries Service, Office of Protected Resources, Silver Spring, Maryland, United States of America; 7 U.S. Geological Survey, Wetland and Aquatic Research Center, Davie, Florida, United States of America; MARE – Marine and Environmental Sciences Centre, PORTUGAL

## Abstract

Marine Spatial Planning (MSP) provides a process that uses spatial data and models to evaluate environmental, social, economic, cultural, and management trade-offs when siting (i.e., strategically locating) ocean industries. Aquaculture is the fastest-growing food sector in the world. The United States (U.S.) has substantial opportunity for offshore aquaculture development given the size of its exclusive economic zone, habitat diversity, and variety of candidate species for cultivation. However, promising aquaculture areas overlap many protected species habitats. Aquaculture siting surveys, construction, operations, and decommissioning can alter protected species habitat and behavior. Additionally, aquaculture-associated vessel activity, underwater noise, and physical interactions between protected species and farms can increase the risk of injury and mortality. In 2020, the U.S. Gulf of Mexico was identified as one of the first regions to be evaluated for offshore aquaculture opportunities as directed by a Presidential Executive Order. We developed a transparent and repeatable method to identify aquaculture opportunity areas (AOAs) with the least conflict with protected species. First, we developed a generalized scoring approach for protected species that captures their vulnerability to adverse effects from anthropogenic activities using conservation status and demographic information. Next, we applied this approach to data layers for eight species listed under the Endangered Species Act, including five species of sea turtles, Rice’s whale, smalltooth sawfish, and giant manta ray. Next, we evaluated four methods for mathematically combining scores (i.e., Arithmetic mean, Geometric mean, Product, Lowest Scoring layer) to generate a combined protected species data layer. The Product approach provided the most logical ordering of, and the greatest contrast in, site suitability scores. Finally, we integrated the combined protected species data layer into a multi-criteria decision-making modeling framework for MSP. This process identified AOAs with reduced potential for protected species conflict. These modeling methods are transferable to other regions, to other sensitive or protected species, and for spatial planning for other ocean-uses.

## Introduction

Marine Spatial Planning (MSP) provides a solutions-focused pathway for ocean planning through the use of spatial data and models to evaluate interactions across multiple spatial and temporal scales. An MSP approach provides opportunities to evaluate tradeoffs among environmental, social, economic, cultural, and management considerations to inform how and where ocean and coastal industries can be sited (i.e., strategically located). Ecosystem-level MSP (i.e., planning at the regional or ocean basin scale) presents unique challenges where expanded datasets and broader scale determinations are required and data are often limited. MSP approaches are being implemented throughout the world to determine space appropriate for pioneering ocean industries (e.g., wind energy, aquaculture) [1, 2]. Finding the most suitable ocean space for emerging industries is challenging, given historic and current ocean uses and the potential for disrupting established industries that could have global socioeconomic impacts. Introducing new or additional anthropogenic stressors to an ecosystem requires appropriate consideration of cumulative impacts from industries, especially in the context of environmental change (e.g., climate change). MSP is a useful tool for regulators and scientists to make informed and transparent decisions that balance the demand for ocean-based food and energy production with potential ecosystem or resource impacts.

Aquaculture is the fastest growing food sector in the world; however, only seven percent of the United States seafood supply comes from aquaculture [3]. The United States ranks among the top nations in the world in opportunity for offshore aquaculture development given the size of the U.S. economic exclusive zone (EEZ; 3.4 million square miles), the diversity of habitats (polar to tropical), and variety of candidate cultivation species. Innovative farm design and engineering provide aquaculture gear and cultivation approaches that can withstand dynamic offshore conditions and increase production capacity [4–6].

Aquaculture development may impact vulnerable anadromous and marine protected species, including marine mammals, sea turtles, and fishes, that are reliant upon marine habitats (e.g., surface, mid-water, and benthic environments) for survival and reproductive success. Aquaculture development impacts to protected species can vary across a range of activities including environmental and cultural resource surveys, construction, operation and management, and farm decommissioning. Potential impacts can include attraction to farms or displacement from critical habitats which can result in changes to distribution, behaviors, or social structures [7–9]. Physical interactions with gear, vessel traffic, noise, and light pollution can also result in injuries or mortalities [8, 10, 11]. When farms are sited and managed properly, impacts to protected resources can be minimized by avoiding sensitive areas or time periods and using best management practices for construction and operation. Because farms may be developed in areas with existing anthropogenic threats [12], it is important to consider cumulative impacts to protected species as an important siting consideration.

U.S. Presidential Executive Order 13921 (E.O.) (May 7, 2020) called for the expansion of sustainable seafood production in the United States The E.O. required the Secretary of Commerce, in consultation with relevant federal agencies, to identify Aquaculture Opportunity Areas (AOA) suitable for potential commercial offshore aquaculture development. Under the E.O., AOAs should use comprehensive data acquisition, novel modeling methodologies, and public stakeholder engagement to support environmental, economic, and social sustainability; and minimize resource use conflicts. To support the E.O., the National Oceanographic and Atmospheric Administration (NOAA)’s National Centers for Coastal Ocean Science (NCCOS) commenced an MSP study to identify suitable AOA options in federal waters of the U.S. Gulf of Mexico and southern California [13, 14].

Under this E.O. directive and following the legal mandates of the U.S. Endangered Species Act (ESA) (16 USC. 1531 et seq.) and Marine Mammal Protection Act (MMPA) (16 USC. § 1361) to avoid or minimize effects to protected species, we developed a generalized approach to integrate pertinent marine protected species data into the AOA MSP model. In each region, AOA options that minimize potential interactions with vulnerable species and critical habitats for reproduction, feeding, migration, and residency were identified. Although specific to planning for AOAs, this standardized integration of transient protected species data into a multi-criteria decision-making MSP modeling framework is transferable to assess the potential risk of other protected species and ocean-use conflicts. The main goal of this study is to provide generalized guidance for avoiding development conflicts with protected species in the marine environment. Here, a case study is provided for an MSP modeling process for AOAs in the U.S. Gulf of Mexico.

## Methods

### Generalized scoring for protected species data

In the NCCOS MSP approach [13], each discrete cell within subregions is assigned a suitability score for a proposed activity. These scores range from 0 to 1 for each available data layer. A score of 1 reflects an area of highest “suitability” (i.e., no siting conflicts), and a score of 0 reflects an area that is unsuitable for the proposed activity. A generalized score of 0.5 was reserved for layers or areas within layers to reflect types of data needing further consideration during the site characterization process. The generalized score is the standard score used in the spatial model for data where suitability or compatibility of a location for potential activities is uncertain. Ultimately, scored data layers are equally-weighted and combined using a geometric mean to determine the overall “suitability” of a given cell for the proposed activity.

To categorize protected species siting conflicts within the constraints of this generalized scoring system, we assigned scores to ESA-listed species and MMPA stocks ranging from 0.1 (most vulnerable species, based on their biological status) to 0.8 (least vulnerable species), based on the best available data for each protected species considered (**Table 1**). This generalized scoring system provided relative measures of protected species vulnerability based on species status under the ESA or MMPA, population size, and population trajectory, as determined from stock assessments [15] or the NOAA Fisheries Report to Congress [16], to inform relative risk in spatial modeling (**Table 1**). In this system, ESA-listed species and MMPA stocks are ranked according to factors that are more or less likely to affect their ability to withstand mortality, serious injury, or other impacts to the species’ ability to survive and recover. Because specific aquaculture activities are not identified upfront with the creation of an AOA, a generalized approach was desired that could universally provide a general, comparable measure of protected species risk across aquaculture types and regions. In the United States, where protected species status and trend are required assessments with clear guidelines (84 FR 18243) under the ESA and MMPA, scoring can be easily determined by referencing stock assessments or Reports to Congress [15, 16]. The generalized approach of scoring species based on relative differences in statutory protection, status, and trend is consistent with NOAA’s Congressional reporting through the Government Performance and Results Act (GPRA) process (5 U.S.C. 306 Chapter 3).

**Table 1:** Generalized scoring system for protected species.

Status	Trend	Converted scores for model
**ESA Endangered **	declining, small population^a^ or both	0.10
**ESA Endangered **	stable or unknown	0.20
**ESA Endangered **	increasing	0.30
**ESA Threatened **	declining or unknown	0.40
**ESA Threatened **	stable or increasing	0.50
**MMPA Strategic**	declining or unknown	0.60
**MMPA Listed**	small population*	0.70
**MMPA Listed**	large population	0.80

A generalized scoring system for Endangered Species Act (ESA)-listed species and Marine Mammal Protection Act (MMPA)-listed stocks. A stock is defined by the MMPA as a group of marine mammals of the same species or smaller taxa in a common spatial arrangement that interbreed when mature.

^a^Small population equates to populations of 500 individuals or less [17].

### Combining protected species data layers

To determine the most conservative approach providing the highest contrast in the AOA model, four mathematical approaches to combining scores across protected species data layers to generate a final combined protected species data layer were evaluated: 1) Geometric Mean (*g*) (Eq 1), 2) Arithmetic Mean (*μ*) (Eq 2), 3) Product (*ρ*) (Eq 3), and 4) Lowest Scoring layer (*l*) (Eq 4). These are computed at the model cell level as follows:

$$g=\sqrt[{n}]{x_{1}\times x_{2}\times \ldots \times x_{n}}$$ Eq 1

$$\mu =\frac{x_{1}+x_{2}+\cdots +x_{n}}{n}$$ Eq 2

$$\rho =x_{1}\times x_{2}\times \ldots \times x_{n}$$ Eq 3

$$l={min(x}_{1},x_{2},\ldots ,x_{n})$$ Eq 4

where *x* represents scores based on status and trend (**Table 1**) for species 1 to *n* within a given cell in the model domain.

To evaluate the utility of these mathematical methods to combining generalized scores across species, we developed hypothetical protected species data layers. We considered a geographically-constricted, endangered species with a declining, small population (score = 0.1); a broadly-distributed declining threatened species (score = 0.4); and a broadly-distributed marine mammal with a large population (score = 0.8). Under these hypothetical models of habitat use, cells falling outside the range would be scored a ‘1’ for a given species. We combined the scores across the three hypothetical protected species data layers at the cell level, using the four mathematical methods. We evaluated the performance of the four mathematical methods with regards to directionality of scoring and spread of combined scores. To evaluate directionality, we examined the outcomes of ranking protected species scores on a cell-by-cell basis, with ties ranked by minimum value similar to sports rankings (i.e., 1, 2, 3, 3, 4, 5, 6, 6, 7, etc.). To evaluate spread, we compared boxplots across methods.

### Case study: Gulf of Mexico aquaculture

**Fig 1:**
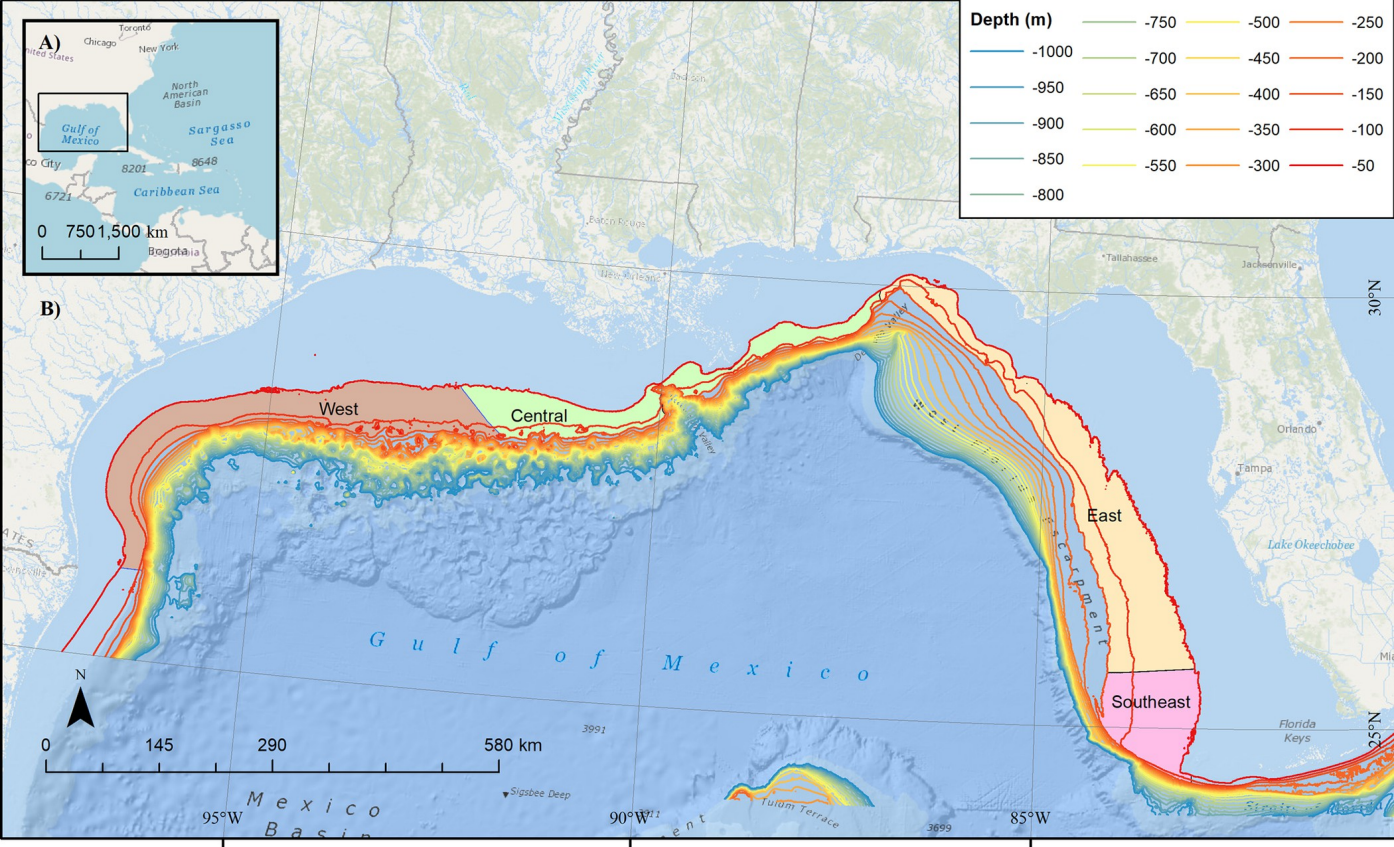
U.S. Gulf of Mexico potential aquaculture opportunity areas. Data were evaluated within 10-ac (4.05-ha) cells within each of four subregions (West, Central, East, Southeast).

NOAA has been tasked with identifying AOAs over seven or more years using the best-available science to identify areas suitable for domestic aquaculture production, while minimizing impacts to fisheries and protected species. To facilitate identifying AOAs, NCCOS began work on a public-facing Atlas [13] to visually represent the relative suitability of different locations for potential aquaculture activities. Through a large interagency consultation process, the study areas for the Gulf of Mexico potential AOA were pre-defined as federal waters between 50–150 m depth across four subregions (**Fig 1**: West, Central, East, and Southeast). The goal of the Atlas was to apply MSP to identify the three most suitable (e.g., least potential conflict) aquaculture opportunity areas in each subregion, ranging from 500–2000 ac (202–809 ha).

**Fig 2:**
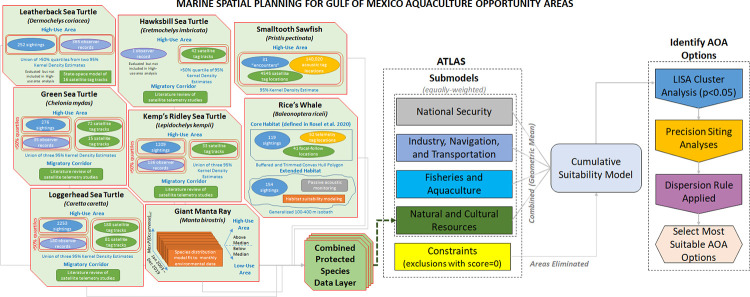
Gulf of Mexico aquaculture marine spatial planning process. Flow chart illustrating how protected species data layers were generated from underlying data and integration of the combined protected species data layer (red outlines) into the broader Aquaculture Opportunity Area (AOA) Atlas marine spatial planning process used to identify final AOA options, as further described in [13].

The Atlas grouped data layers representing aquaculture siting considerations into five submodels: 1) National Security; 2) Industry, Navigation, and Transportation; 3) Commercial Fishing and Aquaculture; 4) Natural and Cultural Resources; and 5) Constraints **(Fig 2**). This modular approach provided a consistent, categorical structure for organizing complex and dynamic ocean data within the planning exercise [18]. The data within submodels were mapped in a Geographic Information System (GIS) at a resolution of 10-ac (4.05-ha) grid cell size and are described comprehensively in [13]. To briefly summarize, the National Security submodel included military operating areas, special use airspace, danger zones and restricted areas, and unexploded ordinance sites. The Industry, Navigation, and Transportation submodel included AIS vessel traffic maps by industry, navigational aids, anchorage areas, oil and gas leases and infrastructure, shipping fairways, and submarine cables. The Commercial Fishing and Aquaculture submodel included commercial shrimp, bandit, bottom longline, menhaden purse seine, and pelagic longline activity as well as headboat fishing and live rock aquaculture locations. The Natural and Cultural Resources submodel included the Flower Garden Banks National Marine Sanctuary and the protected species data layer that is the subject of this case study. The Constraints submodel included activities and occupied areas unsuitable for aquaculture siting (e.g., military zones, coral, and hardbottom; [13]) and were assigned a score of 0. The submodels were combined to form a Cumulative Suitability Model.

As illustrated in **Fig 2**, to inform the Natural and Cultural Resources submodel and effectively capture considerations for multiple, overlapping protected species within the equally-weighted, geometric mean combination scoring scheme of the developing Atlas [13], we evaluated the best scientific information available for the distributions of vulnerable ESA-listed species within the proposed Gulf of Mexico Atlas study areas [19] and scored those layers following the generalized approach described above and presented in **Table 2**. ESA-listed sperm whales were not included because they typically occur in waters deeper than the Atlas study areas [19]. Gulf sturgeon and oceanic whitetip shark were not considered, as they occur inshore and offshore of the proposed study area, respectively. Marine mammals protected solely under the MMPA were not included because insufficient time and resources were available to obtain and evaluate their distributions relative to the proposed Atlas study areas. The species considered (i.e., Rice’s whale, smalltooth sawfish, sea turtles, and giant manta ray) had vastly different types of data available to inform their distribution and the identification of high- and low-use areas. Below, we describe how spatial data were combined to generate scored data layers for each protected species and then combined to create a comprehensive protected species layer for the Atlas study area that could be integrated into the Natural and Cultural Resources submodel. We hope that the detailed presentation of how to arrange different types of available data into a spatially-explicit and meaningful relative scoring scheme will facilitate the adaptation of these methods to other regions and applications.

**Table 2:** Suitability scores for endangered species act listed species occurring in the Gulf of Mexico aquaculture atlas study area.

*Common name*	*Species name*	*Status and Trend*	*Score*	*Source*
*Rice’s whale* ^*a*^	*Balaenoptera ricei*	ESA Endangered, small and declining	0.1	[15]
*Leatherback sea turtle*	*Dermochelys coriacea*	ESA Endangered, declining	0.1	[16]
*Kemp’s ridley sea turtle*	*Lepidochelys kempii*	ESA Endangered, unknown	0.2	[16]
*Hawksbill sea turtle*	*Eretmochelys imbricata*	ESA Endangered, unknown	0.2	[16]
*Smalltooth sawfish*, *U*.*S*. *DPS*	*Pristis pectinata*	ESA Endangered, increasing	0.3	[16]
*Giant manta ray*	*Manta birostris*	ESA Threatened, declining	0.4	[16]
*Loggerhead sea turtle*, *Northwest Atlantic Ocean DPS*	*Caretta caretta*	ESA Threatened, unknown	0.4	[16]
*Green sea turtle*, *North Atlantic DPS*	*Chelonia mydas*	ESA Threatened, increasing	0.5	[16]

The eight ESA-listed species known to occur within the Gulf of Mexico Aquaculture Atlas study areas and their suitability scores, as determined by species status and trend. Note: ESA-listed corals are not included because areas containing corals are scored as ‘0’ (not suitable for aquaculture activities) for the Atlas.

^a^Formerly Gulf of Mexico Bryde’s whale (*Balaenoptera edeni*)

#### Rice’s whale

This study uses a “core habitat” data layer for Rice’s whale (*Balaenoptera riceii*) defined in [20, 21] using the best available tag and sightings data available as of June 6, 2019 compiled in ArcGIS 10.8.1 (ESRI, Ltd.). For clarify, we briefly summarize the core habitat development method here. A convex hull polygon was developed around all recorded northeastern Gulf of Mexico baleen whale (Rice’s whale, Rice’s/Sei, Rice’s/Sei/Fin) sighting locations (n = 119) from surveys from 1989–2018, telemetry tag locations (n = 52) from a single Rice’s whale tagged in 2010, and focal-follow sighting locations (n = 41) of a whale tagged with an Acousonde tag in 2015 [20–22]. Given the limited data available and extremely small population size of this endangered species, a convex hull polygon was used rather than a kernel density estimator, with the intent of encompassing all detections. In addition, the polygon was buffered by 30 km to account for the 10 km strip width of surveys and the approximately 20 km median daily range of movements from satellite tagged animals [20]. This buffered polygon was trimmed on the western-side to the 410 m isobath, determined based on the current deepest known sighting of 408 m [20, 22].

In addition to this core habitat, an extended habitat for Rice’s whale was inferred from sightings data, long-term passive acoustic monitoring (PAM) [23], and habitat suitability modeling [24]. The supporting data for the extended habitat include 1) all recorded Gulf of Mexico baleen whale sighting locations from surveys from 1998–2018 and 2) detections of baleen whale calls in one year of acoustic recordings from passive acoustic moorings deployed in areas of predicted Rice’s whale habitat along the shelf-break from near DeSoto Canyon in the east to offshore of the Flower Garden Banks National Marine Sanctuary (FGBNMS) in the west during 2016–2017 [23]. In August 2017, a genetically verified sighting of a Rice’s whale occurred along the shelf break offshore of Corpus Cristi, Texas. Additionally, stereotypical Rice’s whale long-moan type calls [25, 26] were detected at three of four northcentral/northwestern Gulf sites [25]. Finally, a predictive habitat model was developed across the Gulf of Mexico [24] based on 154 Rice’s whale groups that were sighted on 14 directed and multi-species line transect surveys from 2003–2019. Within a generalized additive model (GAM) framework, sightings per unit effort were evaluated against potential covariates of depth, SST, surface and bottom salinity, SSH, velocity, log chlorophyll-a (Chl-a), and bottom temperature.

#### Smalltooth sawfish, U.S. DPS

Location data for the U.S. Distinct Population Segment (DPS) of smalltooth sawfish (*Pristis pectinata*) were compiled in GIS from three point sources: 1) US Sawfish Recovery Encounter Database [27, 28], 2) Acoustic tag data [29, 30], and 3) Satellite tag data [31]. The US Sawfish Recovery Encounter Database provides data on sawfish observations from 1999–2017; additional data are continually added. The U.S. Smalltooth Sawfish Recovery Team [32] manages the most updated version of domestic sawfish encounter records and shares these with other databases including the International Sawfish Encounter Database (ISED), curated by the Florida Program for Shark Research (FPSR) at the Florida Museum of Natural History on the University of Florida campus [28]. Information from verified sawfish encounter reports is entered into the database and used for monitoring sawfish populations.

Between May 2016 and April 2019, researchers used passive acoustic telemetry and 3 large data sharing networks of VEMCO VR2 (Innovasea, Ltd.) receivers (i.e., iTag, ACT, FACT) to track movements of 43 large juvenile and adult smalltooth sawfish [29, 30]. During this study, 24 females and 19 males were implanted with transmitters with estimated 4- or 10-year battery lives. Additionally, maximum likelihood positioning estimates generated by Wildlife Computers GPE3 positioning software for 15 satellite-tagged sawfish [29–31] were provided by Jasmin Graham (Florida State University, 2021). Smalltooth sawfish point data derived from the three aforementioned sources were merged into a single GIS dataset and filtered to include only locations in the Gulf of Mexico EEZ. A 95% Kernel Density Estimate (KDE) was generated to encompass a smalltooth sawfish high-use area using the *kernelUD* function in the ‘adeHabitat’ [33] package in *R v4*.*1*.*2* [34].

#### ESA-listed sea turtles

Six sea turtle datasets were collated to inform space use of leatherback (*Dermochelys coriacea*), green (*Chelonia mydas*), hawksbill (*Eretmochelys imbricata*), Kemp’s ridley (*Lepidochelys kempii*), and loggerhead (*Caretta caretta*) sea turtles: (1) Aerial survey data from the Gulf of Mexico Marine Assessment Program for Protected Species (GoMMAPPS; [35]) (three surveys from 2017–2018) and National Resources Damage Assessment (NRDA; [36]) (four surveys from 2011–2012) efforts which spanned the northern Gulf of Mexico and covered the aquaculture study area, including observations of 2,253 loggerheads, 1,209 Kemp’s ridleys, 276 greens, and 252 leatherbacks; (2) Residence area locations from multiple satellite telemetry studies [37], including records from 188 loggerheads, 72 greens, 42 hawksbills, and 33 Kemp’s ridleys; (3) State-space distribution model data from 16 Leatherback satellite tag tracks in the northern Gulf of Mexico [38]; (4) Residence area locations from a satellite telemetry study which included 15 adult female green sea turtles (Schroeder B, unpublished data); (5) Sea turtle observations made by fishery observers in the Gulf of Mexico during 2005–early 2020 in the pelagic longline, shrimp, reef fish, gillnet and shark bottom longline fisheries, including observations of 365 incidentally captured leatherbacks, 180 loggerheads, 136 Kemp’s ridleys, 35 greens, and 1 hawksbill (NOAA Fisheries SEFSC, unpublished data); and (6) Residence area locations from 81 adult female loggerheads tracked as part of several satellite telemetry studies [39].

These datasets were analyzed in GIS using ArcGIS 10.4. High-use areas (HUAs) within the Atlas study area were identified as follows. First, residence area locations derived from satellite telemetry data were converted to KDEs using the Kernel Density function in ArcGIS Spatial Analyst (buffered 18.983 km radius) to produce representations of high-use residence areas consistent with those identified in the literature; specifically, the chosen buffer distance produced polygons that were 1,132 km^2^ which was equivalent to the mean size of loggerhead 90 or 95% KDE reported in previous studies [39–41]. Next, separate KDEs were created from the aerial survey and fishery observations. For the fishery observations, we used a search radius of 19 km within the KDE as was used for the telemetry dataset. We used a KDE search radius of 40 km for the observations made during aerial surveys; the increased search radius was used for these data to account for survey transect spacing and sea turtle movements. The resultant density surfaces were classified into quartiles and the upper two quartiles were extracted as HUAs, producing two outputs, one based on aerial survey observation density and another based on fishery observer observation density. Those three polygon outputs were combined with the Atlas polygon to identify portions of the Atlas study area that were HUAs by species for hawksbills, loggerheads, greens, and Kemp’s ridleys. For leatherbacks, only the aerial survey and fishery observer data were used to produce HUAs.

#### Giant manta ray

Using distance-weighting methods to account for individual survey effort, [42] fit a species distribution model (SDMs) to a combined dataset of giant manta ray (*Manta birostris*) sightings generated from 1) Southeast Fisheries Science Center (SEFSC), 2) North Atlantic Right Whale Consortium (NARWC), and 3) New York State Energy Research and Development (NYSERDA) aerial surveys. The best-fitting model with the highest predictive power estimated peak probability of occurrence in areas with warm sea surface temperatures (SST), moderate Z-transformed SST frontal gradients (Front-Z), nearshore and shelf-edge depths, moderate bathymetric slopes, and increasing Chl-a concentrations [42].

Because aquaculture development and associated impacts to protected species may occur on time scales of many months to years, we wanted to capture the distribution of giant manta rays across a broad suite of environmental conditions. To that end, the final [42] combined survey SDM was fit to gridded monthly environmental data from January 2003 to December 2019. Environmental parameters from the following sources were fit to a 10×10 km GIS grid in R by month and year. Depth was assigned to transect segments from the NOAA National Centers for Environmental Information Coastal Relief Model (CRM), which provides 3 arc-second resolution bathymetry for most areas in the study domain. Data gaps were filled with 1 arc-minute resolution bathymetry from the NOAA ETOP01 database using the R ‘marmap’ package [43]. Slope was derived from bathymetry using Spatial Analyst in ESRI ArcMap 10.8.1, with higher-resolution CRM-derived bathymetry and slope retained when available. Satellite observations of daily SST and 8-day averaged chlorophyll-a (Chl-a) were assigned to daily transect segments from the ERDDAP server using the R ‘rerddap’ package [44]. Frontal gradients of SST were computed using the R ‘grec’ package [45, 46]. For each cell, the maximum predicted probability of occurrence across these 204 months was retained. To provide meaningful contrast to inform the Atlas site selection process, several potential cutoffs were evaluated based on quantiles for maximum probability of presence that would receive the Table 1 score of 0.4. Because predictions from the giant manta ray SDM are not normally distributed, the median is the most appropriate measure of central tendency. Areas above the median maximum predicted value were designated as HUAs and assigned a score of 0.4; all other areas received a score of 1 for giant manta ray.

#### Combined Gulf of Mexico protected species data layer

In the Gulf of Mexico Atlas study areas, the Natural Resources submodel (**Fig 2**) contains a National Ocean Service sanctuaries data layer, consisting of only FGBNMS, and the combined protected species data layer described below. The FGBNMS layer is scored as a 0.5 within FGBNMS boundaries (e.g., unknown suitability) and a 1 in all other areas (i.e., “suitable” for aquaculture). Other areas not suitable for aquaculture [e.g., hard bottom and coral habitat areas of particular concern (CHAPCs)] are eliminated through the Constraints submodel from the AOA analysis after the other submodels are combined using a Geometric mean approach.

We compared four approaches to combining protected species data layers: 1) Product, 2) Geometric mean, 3) Arithmetic mean, and 4) Lowest Scoring species in a given cell, using a custom R script. We combined protected species layers within each AOA subregion (i.e., West, Central, East, Southeast) and then merged the four subregions for comparison of scoring approaches, noting that although the combined protected species data layer can be compared between areas, Atlas study areas will be modeled independently and may not be comparable across Cumulative Suitability Models given potential differences in data types between areas.

Because the results of each Cumulative Suitability Model were used to identify the most suitable (top-ranked) clusters of cells to inform the development of preliminary alternatives of the AOA process, the relative ordering of scoring across cells was more important to the final outcome of the model than the actual scores within cells. Rankings were compared at the cell level to determine if rank orders were maintained across methods.

The combined protected species data layer generated using the Product method was integrated into the Natural Resources submodel of the Atlas. Cells in the Constraints submodel were eliminated and the scores across the remaining submodels were equally-weighted and combined at the 10-ac (4.05-ha) grid cell level using a Geometric mean approach, creating a Cumulative Suitability Model with a score for each cell within each study area (i.e., West, Central, East, Southeast). An equally-weighted site suitability model was utilized for the AOA study because multiple types of aquaculture are proposed, making it difficult to compare between potential spatial conflicts. For example, bottom culture techniques and gear may have different requirements than surface culture techniques. Because the final suitability scores compare different types of activities, the overall score does not have an empirical meaning; however, the relative rank of the scores is important to determining which options are relatively more suitable. Therefore, it is important that the ranked scores of the areas in the protected species layer of the Natural Resources submodel are reflective of where managers are more or less concerned with regards to the vulnerability of protected species.

A Localized Index of Spatial Association (LISA) was then implemented on the cumulative suitability output for all study areas to determine statistically significant clusters (p < 0.05), or the grid cells with the highest suitability relative to others, within a given study area [47]. Within the most suitable clusters in each study area, a two-stage precision siting model was then used to identify and rank multiple AOA options within each cluster and then, secondarily, among clusters of the study areas, which were then characterized and used to provide specific details of preliminary options for AOAs (**Fig 2**). The precision siting model [13] was adapted from the Technique for Order of Preference by Similarity to Ideal Solution (TOPSIS) modeling approaches [48–50] to identify the most suitable potential AOA options in each study area. This approach identifies and ranks the ‘suitable’ locations closest to an ideal solution based on distances [51, 52]. The first step in the precision siting model evaluated the most suitable areas identified from the LISA cluster analysis, restricting them to clusters that could accommodate, at minimum, a 500 ac (202.3 ha) square. Locations were then iteratively identified with options supporting areas of 2000 (809.4 ha), 1500 ac (607 ha), 1000 ac (404.7 ha), and 500 ac (202.3 ha). All potential options identified in the first step of the precision siting model were then ranked within clusters to identify the highest suitable option based on proximity to inlets, lowest relative fishing effort, and lowest relative vessel traffic. Finally, the highest scoring options within clusters were compared across clusters using scaled comparisons of distance to inlets, fishing effort, vessel traffic, and oceanographic conditions. Ultimately, the objective of the study was to identify three options or sites per study area (i.e., highest relative suitability scores), allowing for the distribution of options throughout the region. These sites could be included in supplemental studies to further identify aquaculture opportunities or as part of a comprehensive environmental review. These approaches are described in more detail by [13].

## Results

### Combining protected species data layers

**Fig 3:**
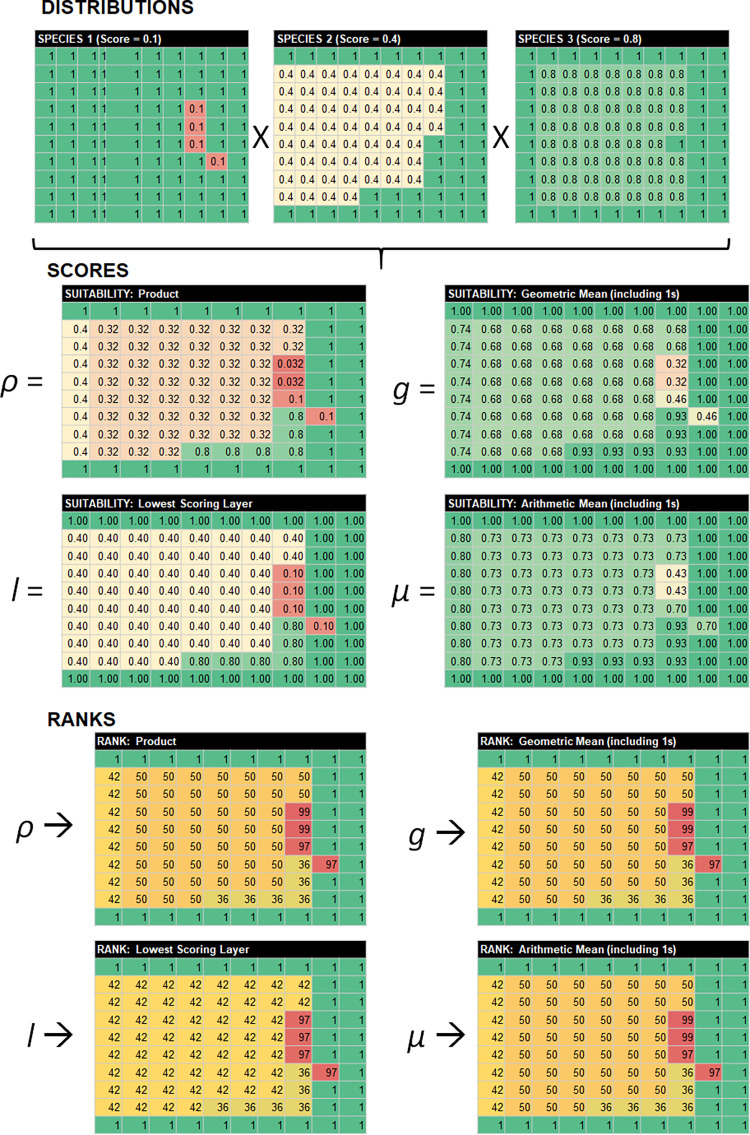
Hypothetical protected species data layers and scoring. Hypothetical combination of three protected species data layers scored following Table 1, using four different approaches: Product (ρ), Geometric mean (g), Lowest Scoring (l), and Arithmetic mean (μ). Warmer colors denote areas of higher concern for protected species.

Using a hypothetical distribution of three species with different distributions, status, and trend (**Fig 3**), we identified issues of concern with regards to applying the Geometric mean, Arithmetic mean, and lowest scoring layer approaches to combined ranked data layers (e.g., **Table 1**). For categorical scores (0/0.5/1) we anticipate the Geometric mean works correctly because a 0 in a cell results in an overall score of 0, and more overlapping cells at 0.5 results in a cumulative lower score without a relative value system imposed. However, when multiple protected species layers overlap and a relative value scale is imposed on those layers, both the geometric and Arithmetic mean methods result in final scores within individual cells that can be higher than the score for the species of greatest concern within the cell. For example, using the hypothetical distributions in **Fig 3**, the score for cells containing Species 1 is higher than 0.1 in both the arithmetic and Geometric mean approaches, because those cells also contain Species 2 and/or Species 3. Also of concern, as more layers are incorporated with relatively limited spatial distributions for the species in question and the remaining areas are scored as 1s for that species, the Geometric mean tends towards 1:

$$g(n=3)=\sqrt[{3}]{0.1\times 0.4\times 1}=0.34$$

$$g(n=4)=\sqrt[{4}]{0.1\times 0.4\times 1\times 1}=0.45$$

$$g(n=5)=\sqrt[{5}]{0.1\times 0.4\times 1\times 1\times 1}=0.53$$

…

$$g(n=10)=\sqrt[{10}]{0.1\times 0.4\times 1\times 1\times 1\times 1\times 1\times 1\times 1\times 1}=0.73$$

Therefore, both averaging approaches reduce the spread in the data with regards to differences between cells, and the Geometric mean further compresses the data towards a score of 1 (i.e., no protected species in cell; area suitable for aquaculture). Only the Product approach generates scores below 0.1 (the minimum value for a single species in **Table 1**) when multiple species overlap. Finally, **Fig 3** indicates that the rank order of scoring is consistent for the Product, Arithmetic mean, and Geometric mean approaches, but the Lowest Scoring approach results in more frequent ties and fails to rank areas with different levels of protected species overlap.

### Case study: Gulf of Mexico aquaculture

#### Rice’s whale

**Fig 4:**
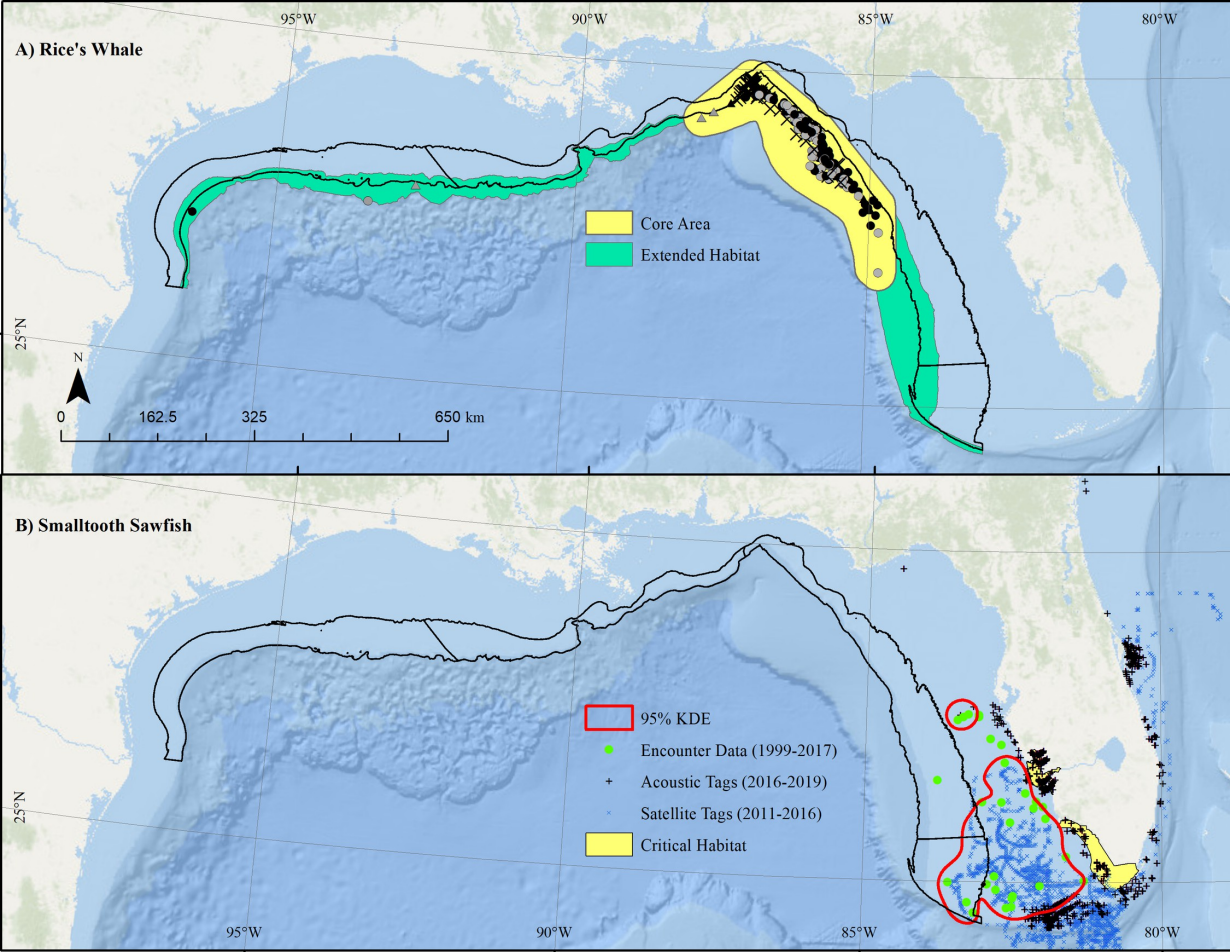
Distribution of Rice’s whale and smalltooth sawfish. Distribution of A) Rice’s whale and B) Smalltooth sawfish relative to proposed Aquaculture Opportunity Atlas study areas (black). Rice’s whale core area (yellow; [20, 21]) distribution polygon based on sightings (black: Rice’s whale, gray: unidentified baleen whale; square: focal follow, triangle: aerial survey, X: satellite tag, circle: ship-based sightin). Extended habitat (green) based on sightings coupled with passive acoustic monitoring (green “extended habitat”). Sawfish high use areas (red line) based on 95% kernel density estimate (KDE) for observations fit to pooled data from Sawfish Encounter Database (1999–2017; ⬤), acoustic tag detections (2016–2019; **+**), and positioning estimates from satellite tags (2011–2016; ×). Note Sawfish critical habitat (yellow) did not overlap the proposed aquaculture area. Map developed in ESRI ArcGIS 10.8.1. Basemap from Esri Ocean Basemap and its partners, reprinted from ESRI under a CC BY license, with permission from ESRI, original copyright 2022. Projection: UTM NAD83 Zone 17N.

Available data on the distribution of Rice’s whale in the northern Gulf of Mexico support a core habitat and an extended habitat (**Fig 4A**). The previously-defined core habitat [20, 21], encompassing the majority of sightings and tag detections, was focused in the northeastern Gulf of Mexico (**Fig 4A**, yellow). Additional sightings, PAM data, and habitat suitability modeling supported an extended habitat (**Fig 4A**, green), representing the distribution of Rice’s whale beyond the core habitat in the western and eastern Gulf of Mexico, roughly tracking the 100–400 m isobaths. The best habitat suitability model, as selected by AIC, included depth, bottom temperature, log Chl-a, and SSH. The model showed good fits to sightings data and low uncertainty within the sampled region. It showed higher uncertainty in deep and shallow waters, but had very low predicted occurrence in those habitats. The habitat suitability modeling process did not consider the PAM data, yet similarly identified a probable distribution along the Gulf of Mexico shelf break concentrated in the core area but extending in a narrow band contained within the 100–400 m depth contours following preferred bottom temperatures along the shelf break throughout the Gulf of Mexico. Thus, sighting data, PAM data, and habitat suitability modeling all supported a core area in the northeastern Gulf and an extended habitat area across the northern Gulf between 100–400 m depth contour with less frequent but year-round occurrences. We assigned a score of 0.1 to the union of the two Rice’s whale layers.

**Smalltooth sawfish, U.S. DPS.** Tagged sawfish were detected off the southeastern United States via 461 VEMCO VR-2 receivers (Innovasea, Ltd.) ranging from off the coast of Brunswick, Georgia, to the lower Florida Keys, and along the Gulf coast to Apalachee Bay, Florida [29, 30]. The areas surrounding Boca Grande, Cape Canaveral, and the lower Florida Keys were the most heavily visited sites (**Fig 4B**). The HUA for sawfish identified from the 95% KDE applied to the merged encounter, acoustic tagging, and satellite tagging data was heavily focused on the southwest Florida area (**Fig 4B**, yellow polygons). All cells intersecting this 95% KDE spatial extent received a score of 0.3, while all other cells received a score of 1 (i.e., high suitability) for smalltooth sawfish.

### ESA-listed sea turtles

**Fig 5:**
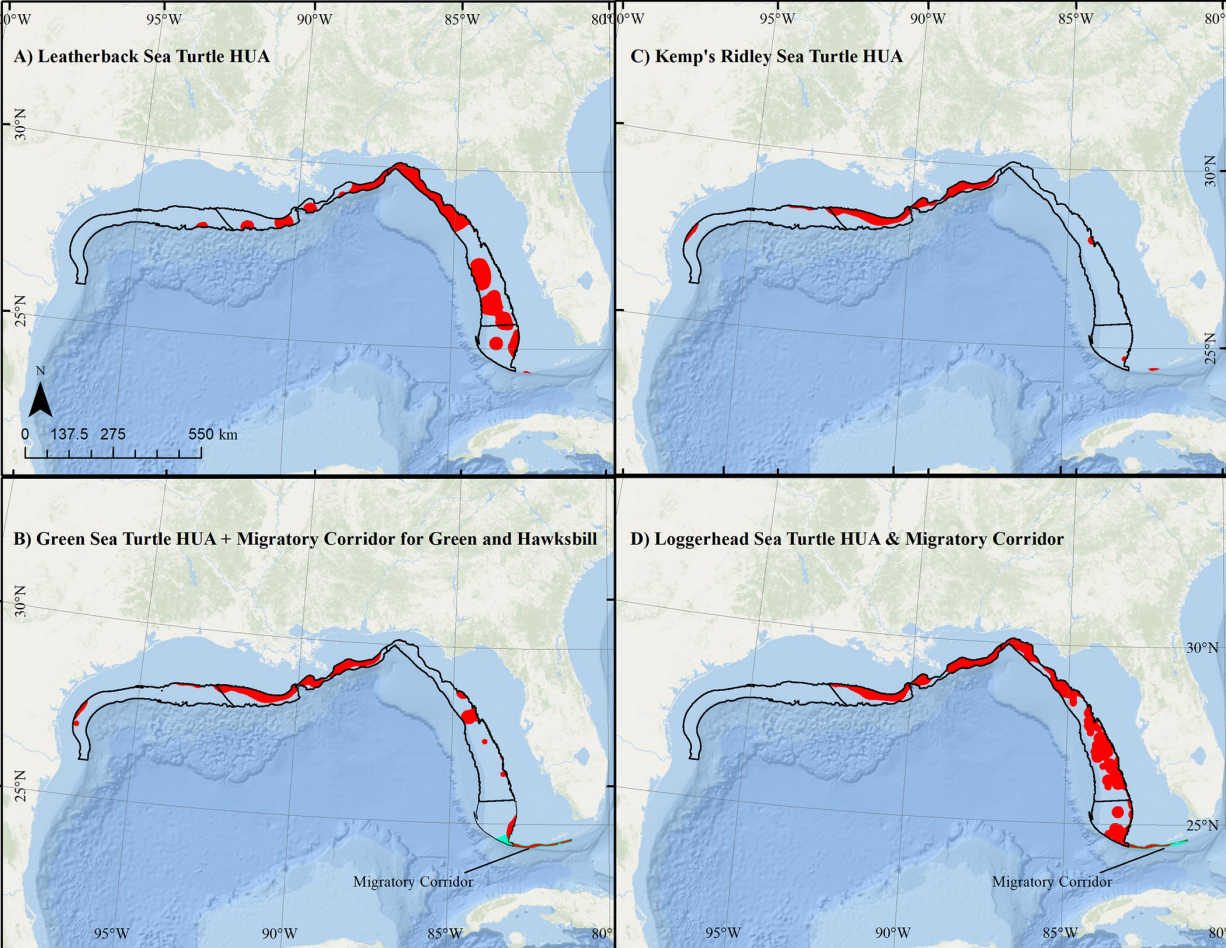
Distribution of sea turtles. High use areas (HUA; red) and migratory corridors (blue) for A) Leatherback sea turtle B) Green and hawksbill sea turtles; C) Kemp’s ridley sea turtle; and D) Loggerhead sea turtles relative to proposed aquaculture study areas (black). Note, due to insufficient data, no high use areas were identified for hawksbill sea turtles within the aquaculture study areas. Map developed in ESRI ArcGIS 10.8.1. Basemap from Esri Ocean Basemap and its partners, reprinted from ESRI under a CC BY license, with permission from ESRI, original copyright 2022. Projection: UTM NAD83 Zone 17N.

Leatherback and loggerhead sea turtle HUAs were identified throughout the northern and eastern Gulf of Mexico, primarily in the deeper habitats of the proposed study areas (**Fig 5A and 5D**). Green and Kemp’s ridley sea turtle HUAs were identified primarily on the inshore edge of the central proposed study area (**Fig 5B and 5C**). Insufficient data were available on hawksbill turtles to identify HUAs using these methods. The southern portion of the Atlas study area, near the Florida Keys, was identified as a high-use migratory corridor for loggerheads, greens, and hawksbills (**Fig 5**) based on a review of previous satellite telemetry studies [37, 53–55]. The union of HUA and migratory corridor layers for leatherback, Kemp’s ridley, hawksbill, loggerhead, and green sea turtles were scored at 0.1, 0.2, 0.2, 0.4, and 0.5, respectively.

#### Giant manta ray

**Fig 6:**
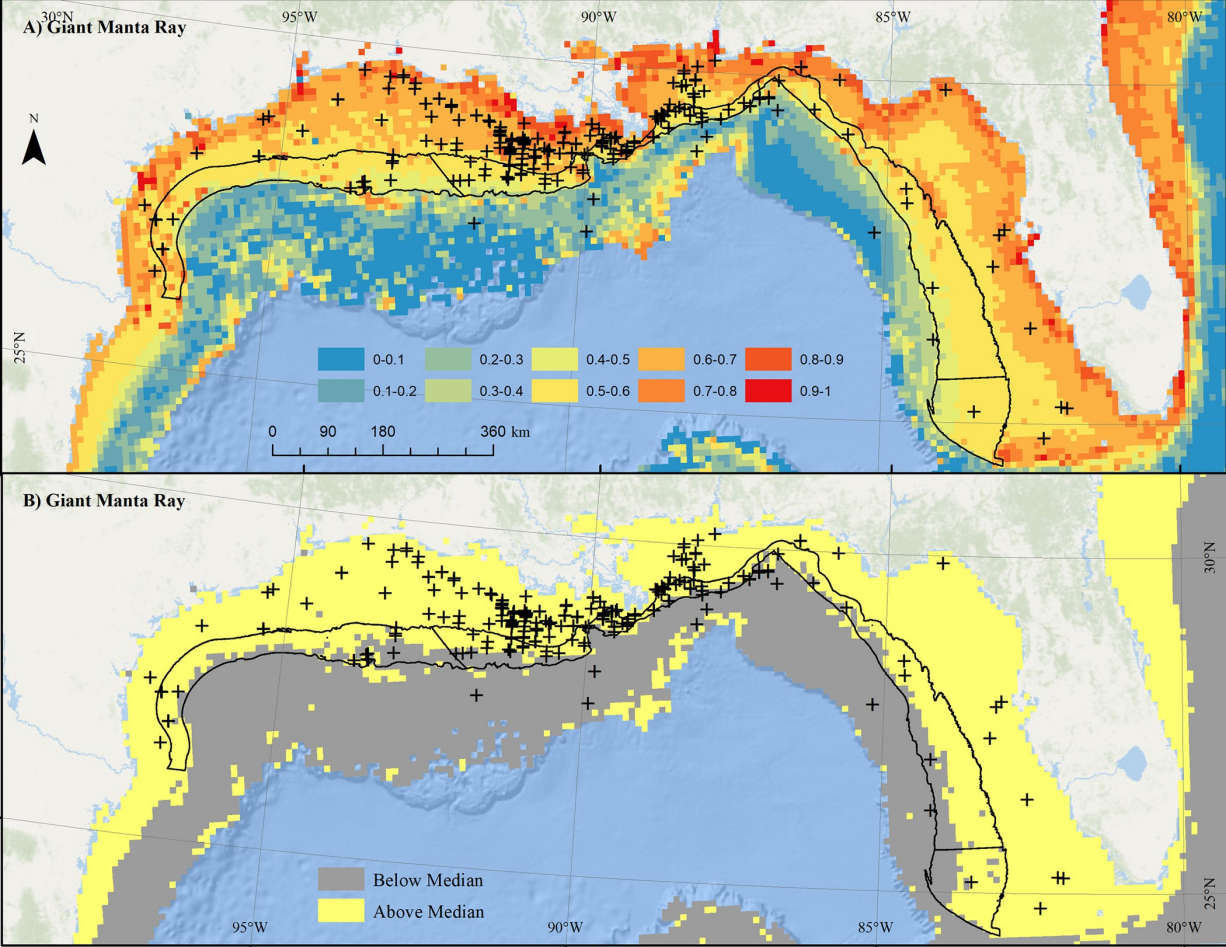
Distribution of giant manta ray. Map of A) maximum monthly predicted probability of giant manta ray observation fit to environmental data from January 2003 to December 2019, overlaid with giant manta ray sightings from 1925–2020, and B) areas falling above (yellow) and below (gray) median predicted probability of occurrence values. Black border denotes proposed aquaculture study areas. Map developed in ESRI ArcGIS 10.8.1. Basemap from Esri Ocean Basemap and its partners, reprinted from ESRI under a CC BY license, with permission from ESRI, original copyright 2022. Projection: UTM NAD83 Zone 17N.

Sightings of giant manta rays in the Gulf of Mexico were heavily concentrated off of Louisiana (**Fig 6**, crosses). Maximum predicted probability of occurrence was highest in coastal environments, especially outside of productive bays and in some shelf-edge environments (**Fig 6A**). Within the proposed aquaculture study areas, maximum probability of occurrence ranged from roughly 0.4 to 1 (**Fig 6A**). Separating giant manta ray scoring at the median probability value resulted in a scored layer (0.4) predominantly on the inshore edge of the aquaculture study areas other than the offshore areas near Flower Garden Banks, southern Texas, and central Louisiana (**Fig 6B**).

#### Combined Gulf of Mexico protected species data layer

**Fig 7:**
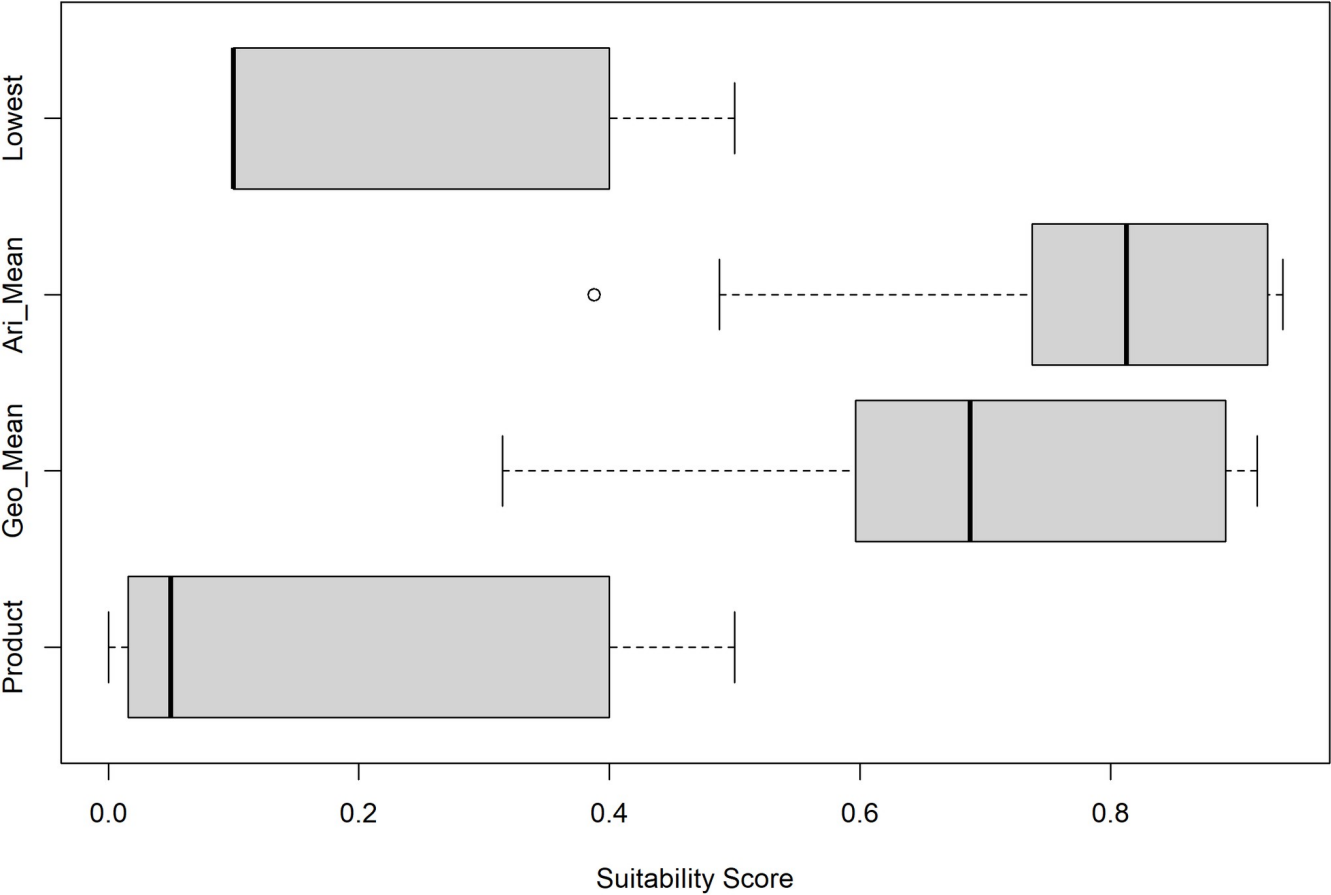
Comparison of scoring methods for combining protected species data layers. Boxplots comparing suitability scores for protected species across the entire Gulf of Mexico study area under Product, Geometric mean, Arithmetic mean, and Lowest Scoring layer methods. Lower scores denote greater vulnerability.

Application of the four scoring approaches to all scored protected species data layers in the Gulf of Mexico proposed aquaculture study areas (**S1 Fig**) provided additional support to the appropriateness of scoring using the Product approach. The resulting scores clearly show the greatest contrast between cells for the Product [median (M) = 0.08, range (R) = 0.000096–1) and Lowest Scoring (M = 0.1, R = 0.1–1) layer approaches, as expressed by both visual spatial contrast between locations (**S1 Fig**) and quantitative analysis of spread in the overall scoring (**Fig 7**). The Arithmetic (M = 0.81, R = 0.39–1) and Geometric mean (M = 0.73, R = 0.31–1) approaches generated the highest overall scores and least contrast between score (**Fig 7**).

**Fig 8:**
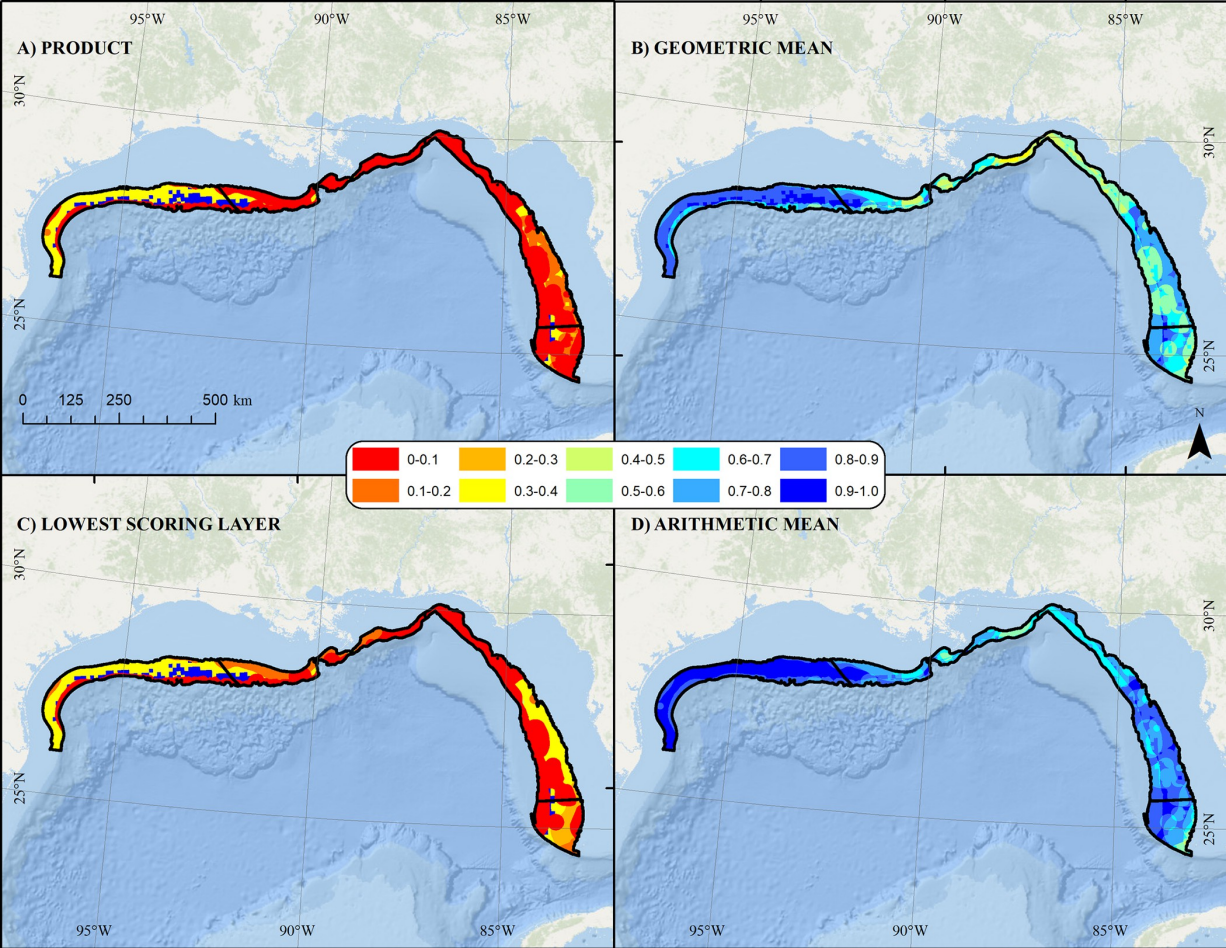
Combined scored protected species data layer. Combined Gulf of Mexico protected species data layer showing A) Product, B) Geometric Mean, C) Lowest Scoring, and D) Arithmetic Mean combined scores across proposed aquaculture study areas (black). Warmer colors denote areas of relatively higher concern with regards to species status, population size, and trajectory. Map developed in ESRI ArcGIS 10.8.1. Basemap from Esri Ocean Basemap and its partners, reprinted from ESRI under a CC BY license, with permission from ESRI, original copyright 2022. Projection: UTM NAD83 Zone 17N.

The Product and Lowest Scoring layer approaches resulted in substantially lower overall aquaculture suitability scores in most areas as compared to the Arithmetic and Geometric mean approaches (**Fig 8**). The final combined protected species data layers using the Product method indicated that high overlap of vulnerable protected species was a concern throughout the Central and East study areas, and also in the furthest offshore areas of the West proposed aquaculture study area (**Fig 8A**). The distribution of Rice’s whale was the primary driver for these outcomes but was further informed by high use areas for several sea turtles and giant manta rays. Similarly, the distribution of smalltooth sawfish was a primary driver for areas of high concern in the nearshore end of the Southeast study area (**Fig 8A**). Both the Product and Lowest Scoring approaches suggested an area of greater aquaculture suitability relative to potential protected species conflicts in the middle of the western study area and in the middle along the border of the eastern and southeastern study areas (**Fig 8A–8C**).

Although the geographic distribution of ranks was relatively similar between the remaining three methods and differences between rankings were mostly subtle (**S2 Fig**), rankings were inconsistent in 93% of cells when comparing between all four methods across all study areas. The Product and Geometric mean approaches provided consistent rankings across all four study areas. The percentage of cells showing differences in rankings between the Product and Arithmetic mean approaches ranged from 15% in the western study area to 87% in the eastern study area. The Lowest Scoring approach was the least consistent with regards to rankings of cells, presumably because it, by definition, did not account for overlap of multiple species. The percentage of cells showing differences in rankings between the Product and the Lowest Scoring approach ranged from 84% in the western study area to 99% in the eastern study area, respectively.

**Fig 9:**
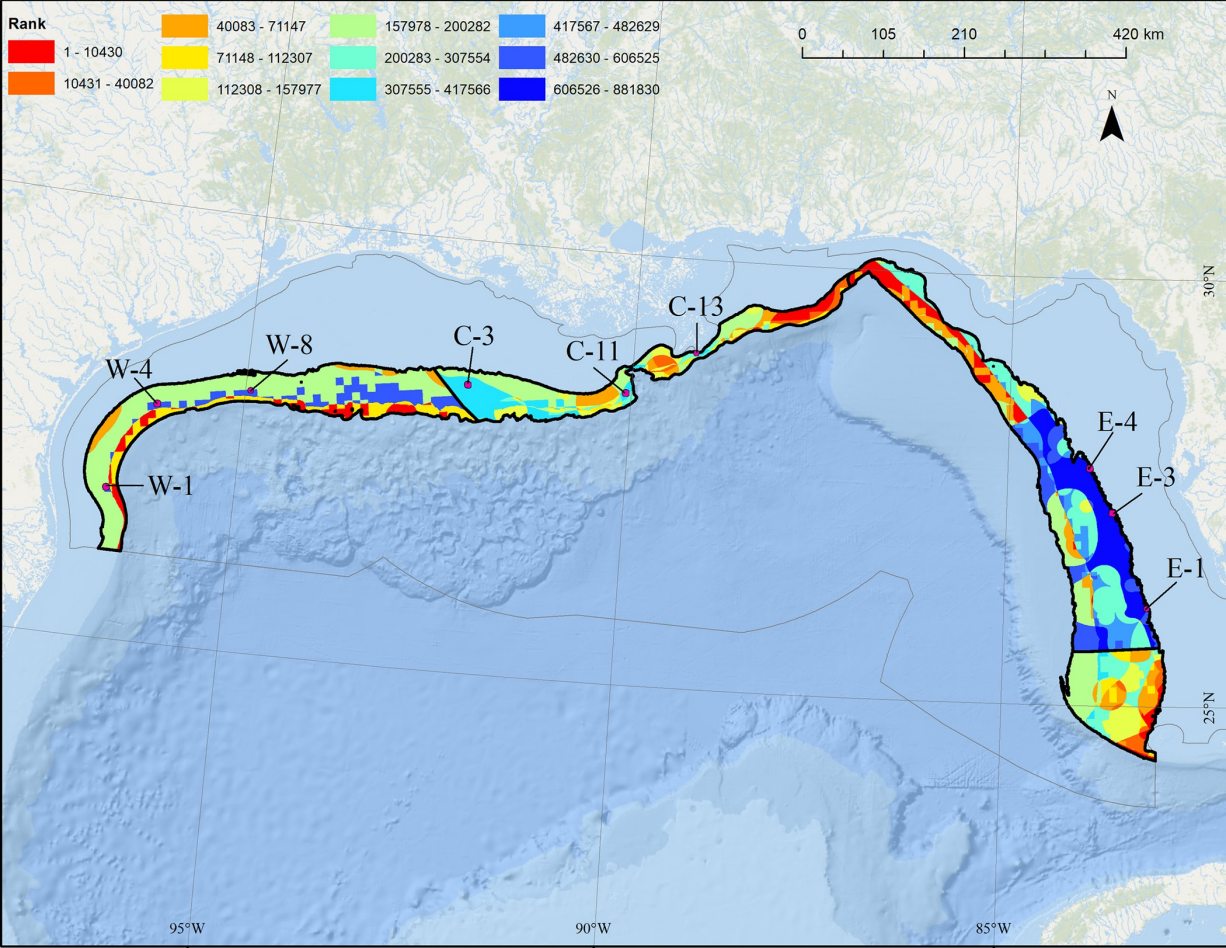
Combined ranked protected species data layer. Ranked scores using Product method relative to the nine final AOA options (green boxes; i.e., W-1, W-4, W-8, etc.) resulting from the AOA analysis (see Fig 1) within each of the four aquaculture study sub-areas (black). Layer generated by combining Rice’s whale, five species of sea turtles, smalltooth sawfish, and giant manta ray layers using the Product method. Warmer colors denote areas of relatively higher concern with regards to species status, population size, and trajectory. Map developed in ESRI ArcGIS 10.8.1. Basemap from Esri Ocean Basemap and its partners, reprinted from ESRI under a CC BY license, with permission from ESRI, original copyright 2022. Projection: UTM NAD83 Zone 17N.

The decision to evaluate four separate study areas provided more relative contrast between cells (**Fig 9**). In the West study area, the area of least concern was in the middle of the proposed aquaculture study area off the border of Texas and Louisiana (**Fig 9**). In the Central study area, areas off western Louisiana were of lower concern than areas off Alabama and Florida (**Fig 9**). In the East study area, only a few smaller nearshore areas were of lower concern, but still generally high concern relative to other areas in the Gulf of Mexico (**Fig 9**). In the Southeast study area, shallower areas were of higher concern (**Fig 9**).

The outcomes of the LISA clustering and precision siting analysis are described in detail by [13]. Briefly, in the West study area, several parcels were identified as highly suitable within the middle depths of the study area between 92°–94° W (**S3 Fig**). In the Central study area, most suitable parcels were identified by the cluster analysis in the middle and offshore depths of the western edge of the study area, between 90°–92° W (**S4 Fig**). In the East study area, most suitable parcels were identified by the cluster analysis in the nearshore edge of the study area off Tampa Bay, between 27°–29° N (**S5 Fig**). Ultimately, due to conflict with National Security space requirements, no suitable areas in the targeted >500 ac (>202.3 ha) size range could be identified in the southeastern study area [13].

Of the 29,839 AOA options from the West, Central, and East study areas, the top nine ranked options (three per study area) were selected for characterization (**Fig 9**, violet boxes). Overall, despite being equally weighted with considerations from the National Security; Industry, Navigation, and Transportation; and Fisheries and Aquaculture [13], the inclusion of the combined protected species data layer developed using the Product method resulted in nine final AOA suitability options that largely avoided areas of high importance for protected species. **Table 3** provides a listing of all protected species data layers included in the combined model used as input to the Natural and Cultural Resources AOA submodel. Of the 108 potential siting conflicts between the 12 separate protected species data layers and the top nine AOA alternatives, only 10 (9.2%) potential siting conflicts occurred, eight of which were related to the broadly-distributed giant manta ray. When excluding the giant manta ray, only three (2.7%) potential siting conflicts remained (loggerhead and green sea turtle).

**Table 3:** Potential siting conflicts with protected species from final aquaculture opportunity area options.

	West Study Area			Central Study Area			East Study Area			
Species Data Layer	W-1	W-4	W-8	C-3	C-11	C-13	E-4	E-3	E-1	
Rice’s whale core distribution area	No	No	No	No	No	No	No	No	No	
Rice’s whale suitable habitat		No	No	No	No	No	No	No	No	No
Leatherback sea turtle high use area		No	No	No	No	No	No	No	No	No
Loggerhead sea turtle high use area	No	No	No	No	No	No	No	**Yes**	**Yes**	
Loggerhead sea turtle migratory corridor		No	No	No	No	No	No	No	No	No
Hawksbill sea turtle high use area		No	No	No	No	No	No	No	No	No
Hawksbill sea turtle migratory corridor		No	No	No	No	No	No	No	No	No
Kemp’s ridley sea turtle high use area		No	No	No	No	No	No	No	No	No
Green sea turtle high use area	No	No	No	No	No	No	**Yes**	No	No	
Green sea turtle migratory corridor		No	No	No	No	No	No	No	No	No
Giant manta ray upper modeled distribution	No	No	**Yes**	**Yes**	**Yes**	**Yes**	**Yes**	**Yes**	**Yes**	
U.S. DPS Smalltooth sawfish high use area	No	No	No	No	No	No	No	No	No	

Top AOA options and interaction (yes/no) with protected species layers used to generate the combined protected species data layer provided to the Gulf of Mexico Aquaculture MSP process. Note the low level of interaction with protected species. Interactions in bold and shaded in gray.

## Discussion

Considerations for stationary protected resources, such as seagrass, corals, or marine protected areas, are often included in MSP models for aquaculture [1, 56, 57]. However, mobile or transient protected resources, such as marine mammals, are generally excluded from MSP. For mobile species, there is often uncertainty as to the impacts of ocean industries during the early planning stages, coupled with uncertainty regarding species distributions, sparse location data, or highly variable location data. However, in some cases, sufficient data are available for summary and integration into an MSP modeling approach. Early integration into planning processes reduces the likelihood of future conflict. Transparency about potential conflicts in the early planning stages can also help avoid contentious and time-consuming permitting decisions during the project design and implementation phase. In this study, we demonstrate how different forms of data for mobile protected resources may be summarized to inform distributions. We integrate across protected species scoring systems and layers using a generalized MSP approach that is portable across species (see **Table 1**) and planning considerations. We identify the Product method as the most appropriate approach for combining data layers that have an internally consistent ranking scheme. Finally, we demonstrate the successful application of this approach to MSP for within the area under consideration for federally-permitted Gulf of Mexico aquaculture.

Spatial data from megafauna is used regularly by managers and policy makers to inform decisions regarding regulations and marine protected area boundaries [58]. Tracking data and utilization density of olive ridley sea turtles in Pongara National Park, Gabon, was used for marine park and zone boundary designation [59]. In [60], 36 species distribution layers of seabirds and pinnipeds were combined into a single composite megafauna layer using a weighted Arithmetic mean to inform marine spatial planning around the Falkland Islands. In the presented case study here, eight data layers representing seven species were combined into a single composite layer for use within the suitability model.

The protected species evaluated in this study varied considerably with regards to population status and trend as well as quality and availability of spatial distributional data. In the U.S., species status and trend are assessed annually by NOAA Fisheries for Reports to Congress [15, 16]. Application of this approach could be readily generalized to other areas; the essential aspect of the scoring table (e.g., Table 1) is that it assigns relative vulnerabilities to the species under consideration, with the lowest score assigned to the most vulnerable species. In our application, that was the Rice’s whale, which is the only species of large whale indigenous to the United States [21]. The population is estimated at fewer than 100 individuals, with mean estimates of <50 individuals remaining [21]. The species was listed under the ESA in 2019 and is exposed to a number of threats in the highly industrialized northern Gulf of Mexico, including vessel strikes, interactions with commercial fisheries, and exposure to industrial noise [22, 61]. As a long-lived marine mammal with low reproduction rates and a very small population size, the loss of a single individual could drive the species to extinction [17, 62]. Sightings, PAM, and habitat suitability modeling all suggested a core habitat in the northeastern Gulf of Mexico and an extended suitable habitat within the 100–400 m isobaths throughout the Gulf of Mexico. We assigned the same score (0.1) to both the core habitat and extended habitat as an expression of the precarious status of the population and a reflection of the growing body of evidence supporting the importance of the extended habitat. PAM studies have detected Rice’s whale calls throughout the year, with no evidence of seasonality, on 16% of days at the westernmost site near the FGBNMS as compared to 1% of days at the central site off Eugene Isle, supporting the year-round importance of the extended habitat area [23, 25]. By comparison, long-moan calls are detected more frequently, on 90–100% of days, in the eastern Gulf of Mexico core habitat [26], though effects of site-specific sound propagation, ambient noise levels, and whale calling rates make it difficult to directly infer site-specific density of animals present from acoustic presence data. This highlights the importance of two distinct habitat regions until the distribution and density of this species is better understood. In contrast, a passive acoustic mooring deployed at 800 m depth offshore of Alabama for two years had no detections of Rice’s whale calls (M. Soldevilla, unpublished data), suggesting limited occurrence in deeper waters.

Preliminary spatial density modeling efforts for Rice’s whale based on sightings data identified a relatively high density area ranging from shelf-edge Alabama to southwest Florida, with further suitable habitat in a more narrow shelf-edge strip extending to central Texas to the west and the Florida Keys to the east [63]. This model was based on general habitat features and may not have captured the biological and physical conditions required to support the Rice’s whale population. Updated habitat suitability modeling by [24] followed a similar pattern and suggested the increased concentration of Rice’s whale in the core habitat was explained by notably higher summer Chl-a concentrations in that area as compared to other regions with suitable bottom temperatures. The Rice’s whale core area is characterized by seasonal advection of low salinity, high productivity surface waters, leading to persistent upwelling driven by both local processes (winds) and intrusion of Loop Current features. Rice’s whales are most commonly observed in the mixing area, characterized by intermediate (non-oceanic) Chl-a concentrations, intermediate bottom temperatures, and high salinity bottom water at the boundary between coastal and deep oceanic waters. Other regions in the Gulf have similar bottom temperatures at the shelf-break, but less surface productivity, which may partially explain the less frequent observations of the species in those areas. The areas west of the core distribution are also characterized by much higher levels of shipping activity and noise associated with oil and gas exploration, both of which have been identified as threats to the species and implicated in the possible contraction of their geographic range [61]. Avoiding or minimizing risk to this species from aquaculture activities, particularly risk from entanglement or vessel strike, is essential to their recovery.

Smalltooth sawfish are rays with a long, flat rostrum edged with teeth. Smalltooth sawfish populations declined dramatically during the second half of the 20th century due to habitat loss associated with coastal development and accidental capture in fisheries. They were listed as endangered under the ESA in 2003. Telemetry studies indicated 58% (43% mature; 57% immature) of tagged individuals migrated seasonally, with the remainder being apparent residents of their tagging locations [29, 30]. Tagged juvenile and adult sawfish of both sexes migrated, which indicates that neither sex nor maturity is a predictor of whether a sawfish will migrate or not. Although both coasts of Florida were used for migration, most individuals consistently used the same coast when they migrated. Avoiding or minimizing risk to this species from aquaculture activities, particularly risk from entanglement, obstruction of migratory corridors, or alteration of foraging habitat, is essential to their recovery.

All species of sea turtles in U.S. waters are listed under the ESA, primarily due to global threats including incidental capture in fishing gear (bycatch), illegal harvest, habitat loss, and egg collection for human consumption. Leatherback sea turtles are the largest turtles in the world, with the widest global distribution of any reptile. They are the only species of sea turtle that lacks a hard shell and forage throughout the water column on jellyfish and salps. Green sea turtles inhabit sub-tropical and temperate regions and are unique among sea turtles in that they are herbivorous, foraging largely on seagrass and algae. Hawksbill sea turtles inhabit tropical and sub-tropical waters and forage on sponges following their pelagic stage. Kemp’s ridley sea turtles are found only in the North Atlantic Ocean, primarily in the Gulf of Mexico, and forage mostly on crabs. Loggerhead sea turtles inhabit sub-tropical and temperate regions and forage on benthic invertebrates such as mollusks and crabs. We identified a broad suite of aerial survey, tracking, and observer data that could be used to characterize important areas for sea turtles within the study area. Although we did not include telemetry data for leatherback sea turtles due to timing constraints for acquiring and processing the data, the combined KDEs encompassed the identified HUAs in [38, 64]. For ESA-listed sea turtles and giant manta rays, avoiding or minimizing risk from aquaculture activities, particularly risk from entanglement, obstruction of migratory corridors, or alteration of foraging habitat, will be important to their recovery.

Protected species data are often limited and subject to substantial bias and uncertainty, owing in part to the rarity of the study organisms as well as limitations on resources to study them. Our acoustic and satellite telemetry data were biased in focal species, life stages, and regions. Tagging locations are biased by tag location, size classes capable of carrying tags, and tag retention/track duration. Movements and space use of juvenile animals may differ substantially from those of adults. Fishery observer records were biased to fisheries with observer coverage, and to areas and time periods where fishing occurs. Fisheries may additionally only select for particular life stages, leading to bias in the overall picture of how the species is distributed. Standardized aerial surveys were subject to both perception and availability bias that may be especially problematic for small, cryptic, or diving animals such as sea turtles and manta rays. The SDM for manta rays accounted for these biases and underlying aerial survey effort [42]. Similar efforts to develop predictive models using aerial, ship, and PAM survey data are underway for most protected species. Those results should be incorporated into updates of the protected species data layer to facilitate adaptive management. Similarly, efforts should be taken to incorporate species not considered in the current planning effort, including marine mammals protected under the MMPA. It should also be noted that although oceanic whitetip shark was not considered, several shark species have been observed at aquaculture sites [65, 66], with records of oceanic whitetip sharks at offshore (2000 m depth) Hawaiian aquaculture cages (Y. Papastamatiou, Florida International University, M. Hutchinson, NOAA Fisheries Pacific Islands Fisheries Science Center, 23 Feb 2022, written communication). Changes in distributions due to changes in environmental conditions or animal behaviors should be incorporated in the adaptive process of leasing potential sites.

The general modeling approach used for the Gulf of Mexico Atlas [13] expands on standard methods used by other Multi-Criteria Decision Analyses (MCDAs) for aquaculture. MSP for aquaculture is generally performed using variants of a MCDAs, such as a suitability model. We have observed that few MCDAs or decision support tools for aquaculture generally expand beyond the suitability analysis. Most studies simply aim to identify areas with the greatest extent of relative suitability for aquaculture development [67–69]. Whereas in the AOA analysis, all suitable areas were identified and through the LISA cluster analysis and subsequent precision siting models a number of smaller discrete areas were identified for review [13]. Additionally, the methodology for how suitability calculations are performed will vary by planning objective. In the Gulf of Mexico AOA Atlas [13], an equally-weighted Geometric mean suitability model was determined to be the most representative for identifying suitable areas for general use aquaculture [57, 70, 71]. Having equal weights ensures no differing expert opinions on weighting and no preference is given to one particular type of aquaculture or industry. Additionally, use of the Geometric mean ensures that all variables contribute equally to the suitability score. The Arithmetic mean assumes high suitability scores can compensate for low suitability scores, while the Product approach assumes high suitability scores cannot offset low suitability scores [72].

Both a hypothetical example and a case study application to siting for Gulf of Mexico aquaculture indicated that the Product approach was the most appropriate method for spatially combining overlapping protected species suitability scores (**Table 2**). The Product approach appropriately accounted for overlap between layers (**Fig 3**), provides the correct ordering of the layers (**Fig 3**), and provides contrast between cells (**Fig 6**) that proved informative to the Atlas siting process (**Fig 9**). Although the Geometric Mean provided identical ordering of scores, the spread of the actual scores was substantially narrower in both the Geometric and Arithmetic Mean approaches, which may influence ultimate clustering outcomes under the LISA and precision siting model steps (see **Fig 2**). It is important to note that the combined protected species is a single input to the Natural and Cultural Resources submodel and is combined by Geometric Mean with the other submodels to create the “Cumulative Suitability Model.” As such, the spread of the scores may be extremely important to ensure that the least suitable sites are not selected by the subsequent cluster analysis. Unlike the majority of layers in the Gulf of Mexico Atlas, which are scored as 0/0.5/1 if they are categorical data, the protected species data layers have a relative value scale imposed (0.1–0.8), which, based on our evaluation, makes the Product approach more appropriate, because unlike the Geometric and Arithmetic Mean, it does not compress scores towards 1 (e.g., “suitable for aquaculture). Similarly, unlike the Arithmetic Mean and Lowest Scoring approach, the Product approach retains the correct ordering of cells with regards to areas of highest and lowest concern, and unlike the Lowest Scoring approach, it accounts for overlap of multiple protected species layers.

Given the methods used to generate and score the combined protected species layer generated from the Product method were performed at the scale of the entire Atlas study area, this layer can be used to classify relative vulnerability for protected species both within and across the four sub-regional study areas (i.e., West, Central, East, and Southeast) in the Gulf of Mexico Atlas. In general application, the binning and resolution through which spatial planning options are generated can have a substantial impact on the final outcome [73, 74]. The combined protected species data layer developed by this study would allow evaluation at multiple scales as recommended by [74]. The base resolution of 10-ac (4.05 ha) used for input into the Cumulative Suitability Model was substantially finer-scale than any protected species data layers generated. The final Atlas considered each study area independently because other data sources could not be similarly compared. This resulted in equal numbers of AOA options from each sub-region besides the Southeast study area, which had insurmountable spatial planning constraints with National Security activities. The Gulf of Mexico Atlas used four study area to provide geographic parity across potential aquaculture options; by ranking within instead of across study area, the final recommended study areas did not fully reflect the protected resources finding that potential siting conflicts were generally much higher in the northern and eastern Gulf of Mexico as compared to the western Gulf of Mexico. As such, the final recommended options reflect the tradeoffs involved in a multiagency negotiated marine spatial planning process.

Our generalized scoring approach also did not consider gear-specific risk associated with defined aquaculture activities. This was deliberate, as the Atlas was intended to inform aquaculture planning prior to having defined aquaculture activities proposed. Under the ESA, federal managers are required to avoid, minimize, or mitigate any federally-permitted activity that might jeopardize the continued existence or recovery of ESA-listed species. Our approach provides a transparent public-facing decision-support tool to avoid planning activities in areas with high overlap of vulnerable protected species. As such, the generalized protected species layer is more analogous to a map of consultation risk, with the final product helping to avoid conflict by reducing the likelihood of substantial investment in aquaculture design and development in areas where protected species overlap is high. A general principle of MSP is to preemptively avoid user conflicts through early engagement. Our method provides a transparent process to illustrate where any action requiring federal permitting would face numerous regulatory challenges due to high co-occurrence with vulnerable species.

The application of this spatial modeling approach for protected resources has broad application across all ocean human uses and also for conservation. This approach provides the capability to synthesize across species and habitats providing a holistic perspective at a regional ocean scale and identifies overlapping high-use areas where conservation is most critical when considering multiple species. While this approach was developed to inform development of aquaculture areas, a similar approach could be used for other development efforts including energy extraction, renewable energy (e.g., wind and marine hydrokinetic), shipping and transportation (e.g., development of new or modified shipping fairways), mining, dredging, and other types of activities that may impact protected resources.

This model provides a generalized approach for assessing the relative sensitivity of ocean spaces to development activities such as aquaculture at a regional scale. The application of this model can be useful when planning for changes of existing ocean industries, new pioneering ocean industries, and for conservation. Given the spatially and temporally dynamic nature of ocean space, frequent model updates are likely required to ensure that the most accurate stock assessment data are being considered. Further development of environmentally-driven models similar to the giant manta ray distribution model [42] can provide additional spatiotemporal resolution and predictive capability. These types of models provide four major benefits: 1) they control for bias in observation effort, 2) they can be fit across a broad suite of environmental conditions observed across many years to provide a conservative estimate of long-term impacts, 3) they are adaptive to dynamic ocean conditions, and 4) they allow identification of spatiotemporal environmental windows where likelihood of species presence is low [75]. Environmental windows can be used to minimize adverse effects during the most impactful activities, such as construction. Generalized guidance may require revision for project activities that involve permanent or semi-permanent habitat change, which include most types of fixed-location operations. As additional species distribution models are developed, further study should be given to opportunities to incorporate uncertainty in the MSP process; potentially by using bootstrapped versions of single-species protected species distribution models to generate bootstrapped combined protected species data layers and then bootstrapping the site selection cluster analysis to evaluate overall sensitivity in the final options.

As offshore aquaculture develops, further adaptive management may be possible by incorporating additional protected species considerations similar to the approach of [76]. Given the limited time and resources available to inform the Gulf of Mexico Atlas and the lack of information regarding the likelihood of location, duration, and impact of various stressors associated with different forms of offshore aquaculture development, our approach was necessarily coarse in its resolution. As leasing progresses, each lease will receive fine-scale consideration of the impacts of individual stressors on each individual protected species with regards to their likelihood, severity, and scope, analogous to the Exposure term in [76] or the relative risk assessment presented in [75]. As these analyses are developed, they can be expanded in geographic scope to additionally facilitate precision siting. This could be accomplished both by presenting activity-specific risk maps (e.g., entanglement risk, acoustic exposure risk, ship strike risk) and by generating cumulative risk maps that combine across activity-specific risk maps using appropriate weighting [76]. Ultimately, the population consequences of anthropogenic disturbance [77] might be visualized in a multi-species spatiotemporal framework.

The rapid development of offshore wind energy in the United States and globally is one specific application where this modeling approach could better inform placement of offshore wind farms on both regional and local scales (precision siting). Presently, offshore wind energy development is being planned or underway across five regions of the United States (Northeast, Mid-Atlantic, Gulf of Mexico, South Atlantic, Pacific Coast) with additional regions being considered. Looking ahead to 2050, offshore wind energy development constitutes one of the largest and most rapid consumers of ocean space with some reports suggesting that over 350,000 km^2^ of ocean space will be developed for the production of food and energy by 2050 compared to the approximately 40,000 km^2^ in 2018 (a 9-fold increase) [78]. The advance development of similar or more advanced models (e.g., [76]), preferentially based on species distribution models that are sensitive to environmental parameters, would facilitate site selection to minimize potential risks to protected species at the outset of planning. Given the increasing pressure on ocean ecosystems globally, approaches such as the one presented here can help to ensure conservation of our planet’s most sensitive species and habitats.

## Supporting information

S1 Fig Input scores for all species included in combined protected species data layer. Spatial distribution of input scores for species included in combined protected species data layer. Warmer colors denote species of greater vulnerability based on status and trend.
(TIF)

S2 Fig Comparison of rankings between scoring methods. Comparison of rank order for overlapping protected resource data layers combined using four different approaches. Warmer colors denote areas of greatest vulnerability.
(TIF)

S3 Fig LISA suitability clusters for West study area. Relative rankings of suitability for sufficiently sized parcels identified by LISA cluster analysis within West study area.
(TIF)

S4 Fig LISA suitability clusters for Central study area. Relative rankings of suitability for sufficiently sized parcels identified by LISA cluster analysis within Central study area.
(TIF)

S5 Fig LISA suitability clusters for East study area. Relative rankings of suitability for sufficiently sized parcels identified by LISA cluster analysis within East study area.
(TIF)

S1 File Final combined protected species data layer. GIS shapefile for final combined protected species data layer containing spatially-explicit information on high-use areas for Rice’s whale, smalltooth sawfish, ESA-listed sea turtles, and giant manta ray.
(7Z) Click here for additional data file.
