# Supplementary figures and images for: Modeling protected species distributions and habitats to inform siting and management of pioneering ocean industries: A case study for Gulf of Mexico aquaculture
Source: PLoS One. 2022 Sep 30;17(9):e0267333. doi: 10.1371/journal.pone.0267333 (PMC9524655; doi:10.1371/journal.pone.0267333)

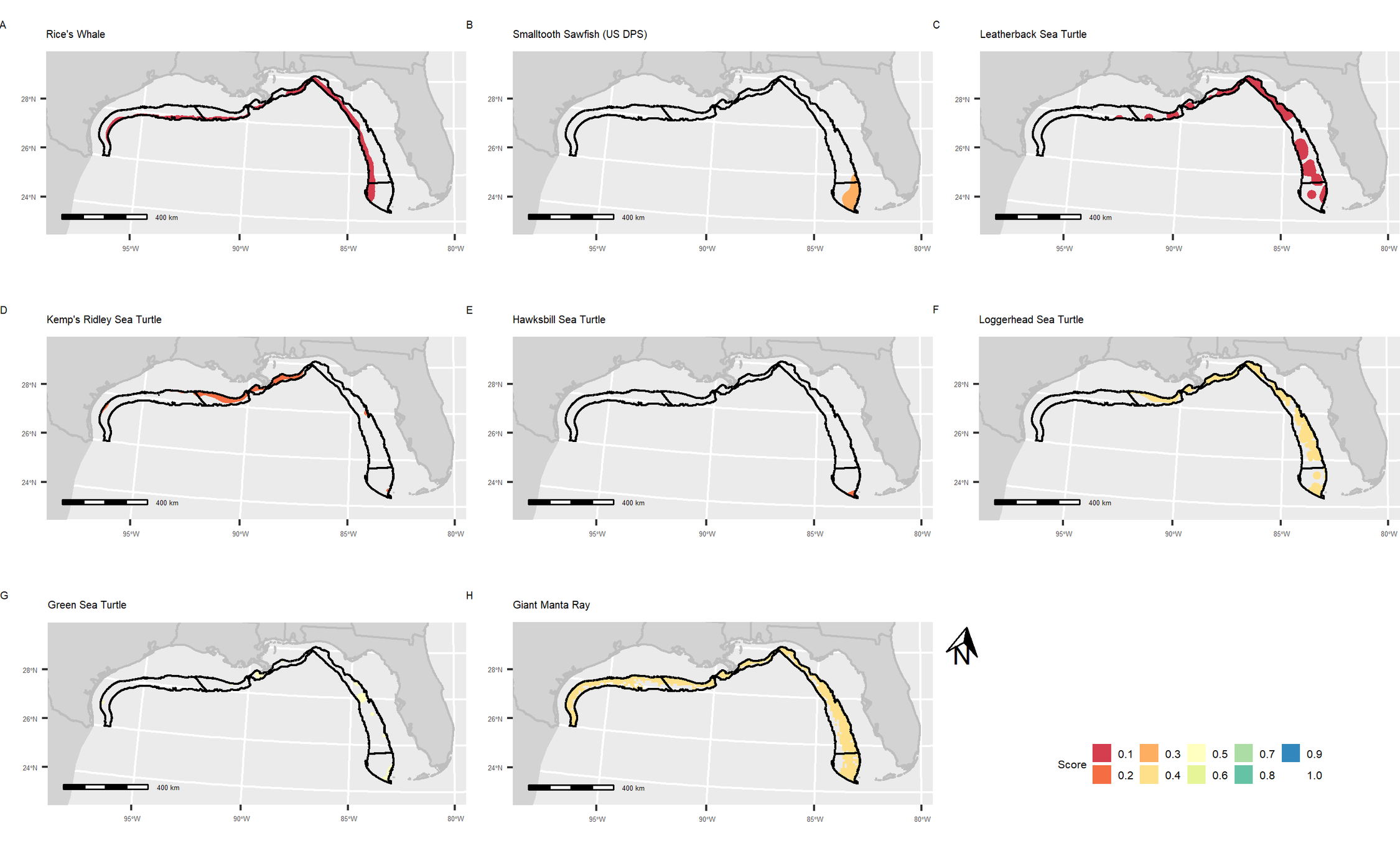

Supplement: S1 Fig — Spatial distribution of input scores for species included in combined protected species data layer. Warmer colors denote species of greater vulnerability based on status and trend. (TIF) [file pone.0267333.s001.tif]

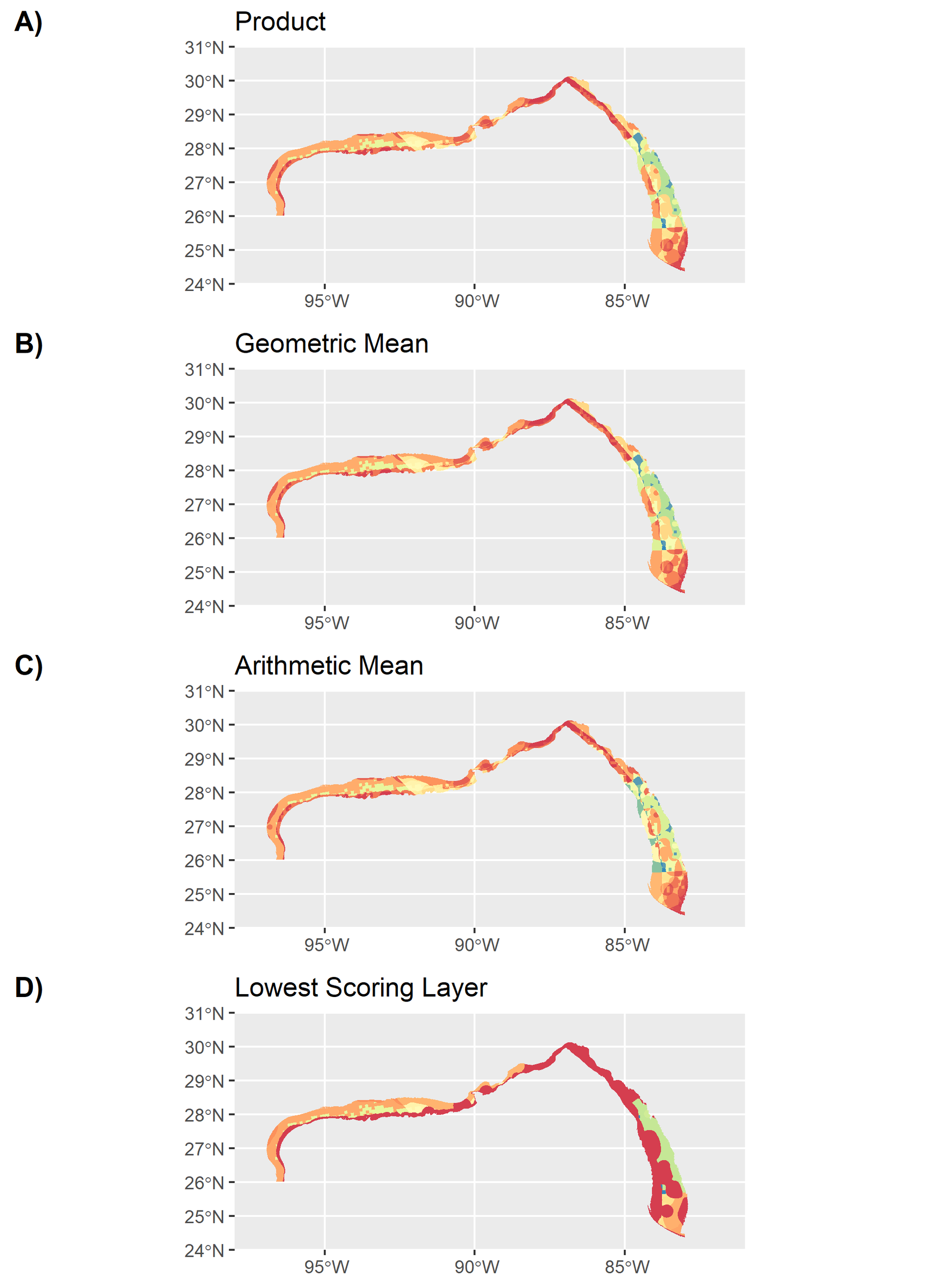

Supplement: S2 Fig — Comparison of rank order for overlapping protected resource data layers combined using four different approaches. Warmer colors denote areas of greatest vulnerability. (TIF) [file pone.0267333.s002.tif]

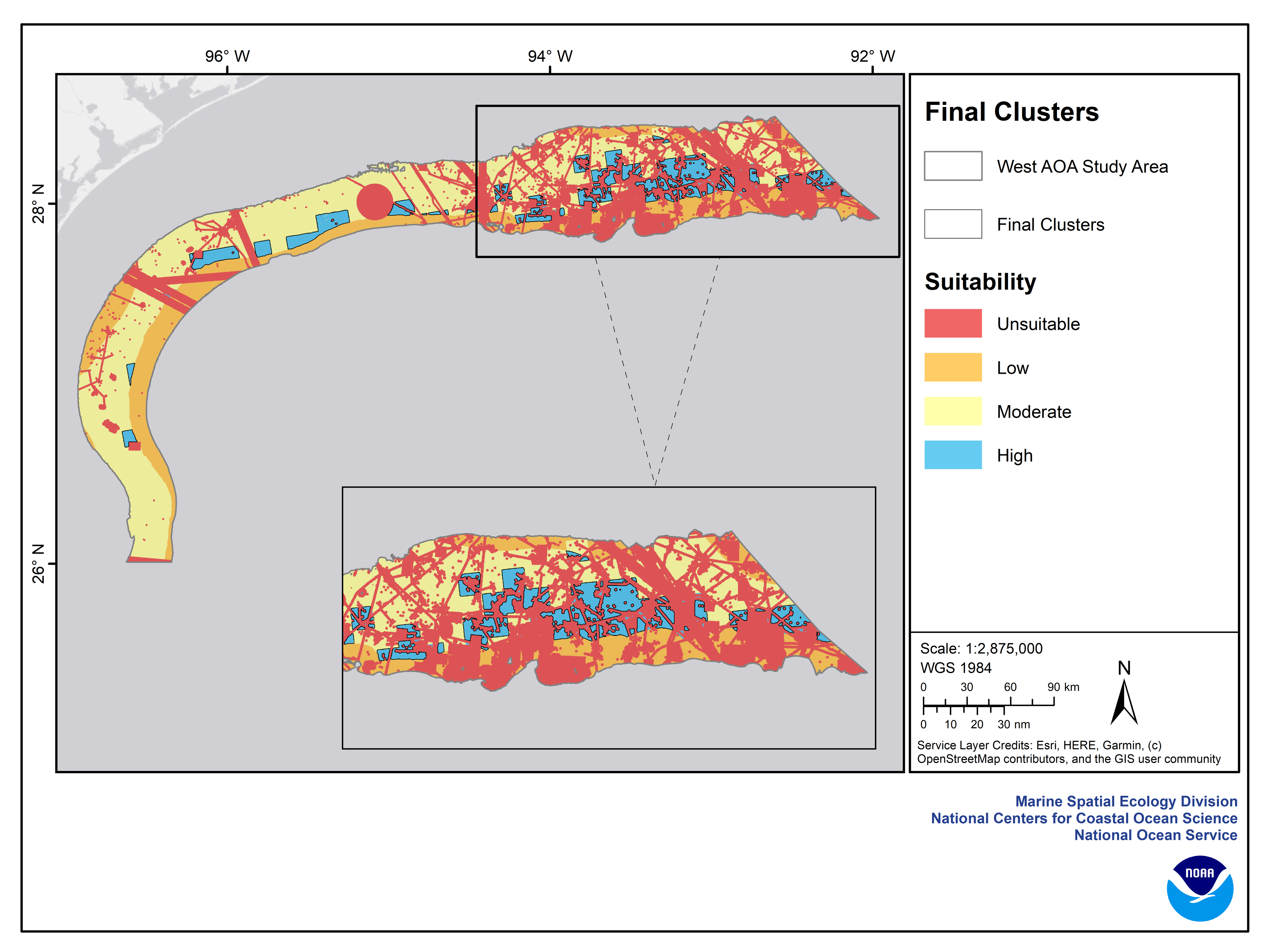

Supplement: S3 Fig — Relative rankings of suitability for sufficiently sized parcels identified by LISA cluster analysis within West study area. (TIF) [file pone.0267333.s003.tif]

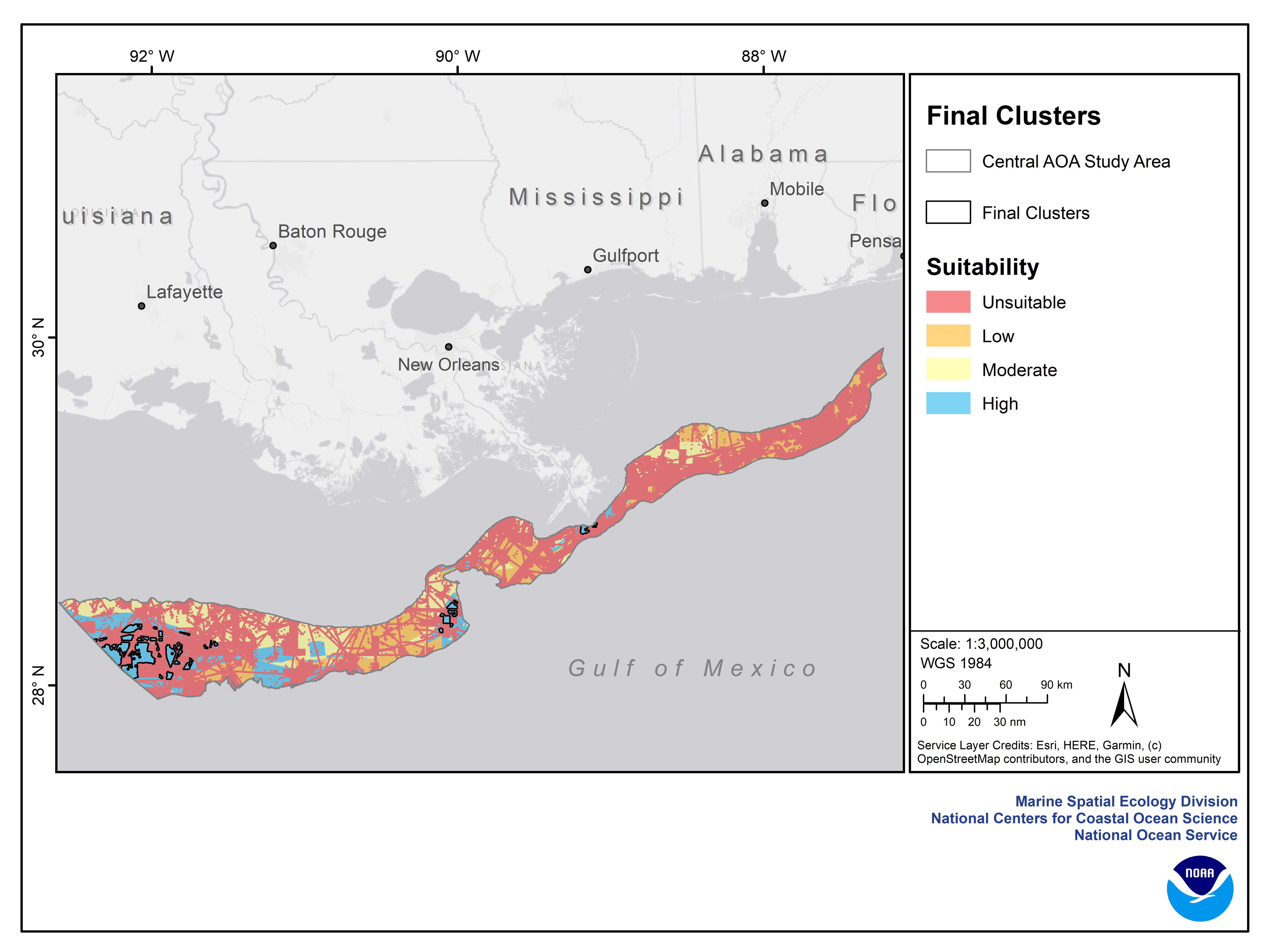

Supplement: S4 Fig — Relative rankings of suitability for sufficiently sized parcels identified by LISA cluster analysis within Central study area. (TIF) [file pone.0267333.s004.tif]

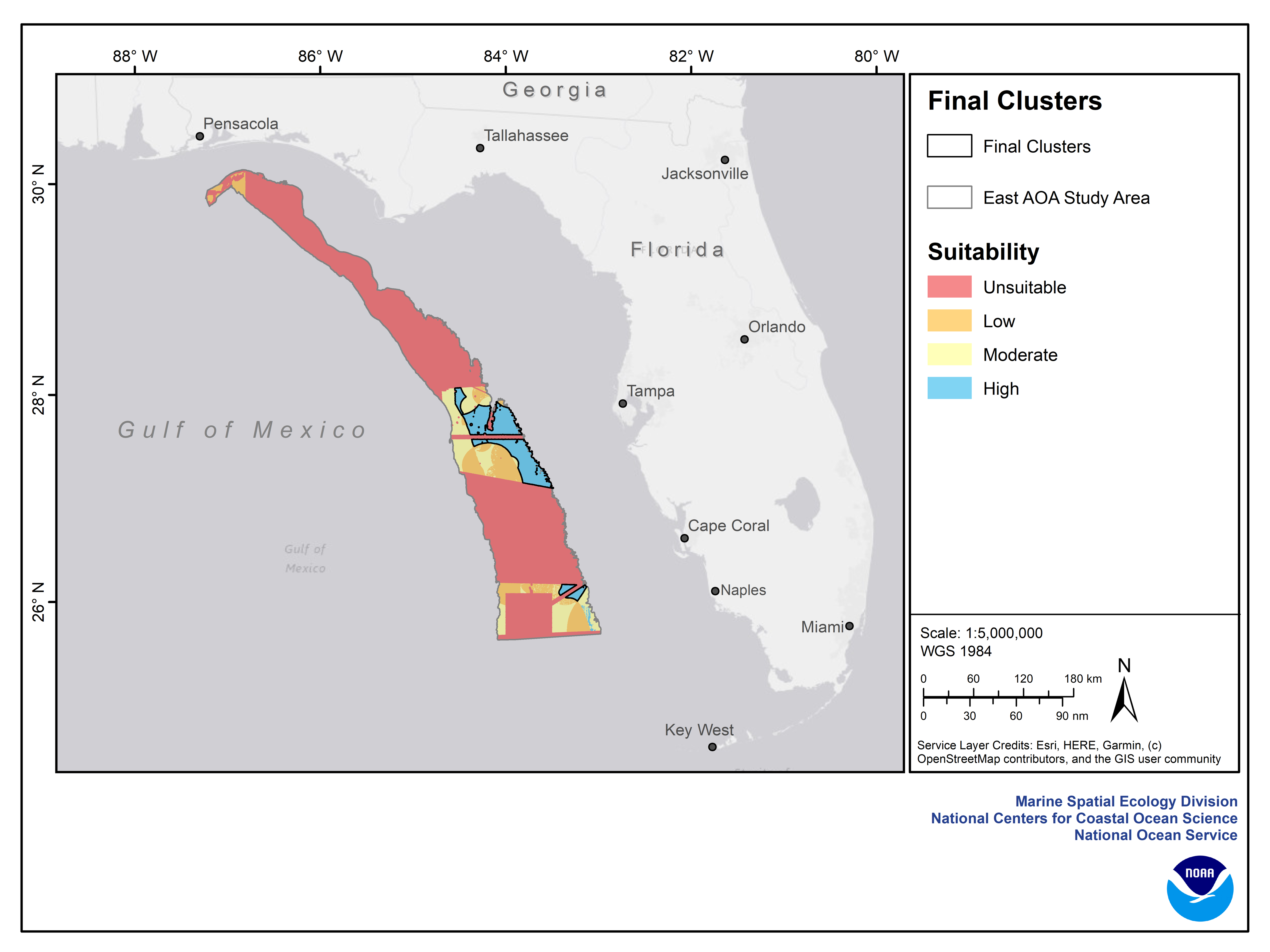

Supplement: S5 Fig — Relative rankings of suitability for sufficiently sized parcels identified by LISA cluster analysis within East study area. (TIF) [file pone.0267333.s005.tif]
